# Mechanical Distention of Organotypic Hippocampal Slice Culture Membranes for High-Throughput *In Vitro* Modeling of Traumatic Brain Injury

**DOI:** 10.1177/2689288X251389788

**Published:** 2025-10-27

**Authors:** Julia Jagielo-Miller, Caleb Bailey, Samir Patel, Paresh Prajapati, Cassidy Leibold, Patrick Sullivan, Mark Prendergast

**Affiliations:** ^1^ Department of Psychology, University of Kentucky College of Arts & Sciences, Lexington, Kentucky, USA.; ^2^ Sanders-Brown Center on Aging, University of Kentucky College of Medicine, Lexington, Kentucky, USA.; ^3^ Department of Physiology, University of Kentucky College of Medicine, Lexington, Kentucky, USA.; ^4^ Spinal Cord and Brain Injury Research Center, University of Kentucky College of Medicine, Lexington, Kentucky, USA.; ^5^ Department of Neuroscience, University of Kentucky College of Medicine, Lexington, Kentucky, USA.

**Keywords:** *in vitro* traumatic brain injury, organotypic hippocampal slice culture, secondary injury, stretch injury, reactive oxygen species

## Abstract

Traumatic brain injury (TBI) results from impact to the head that induces both primary and secondary injuries. Secondary injuries are characterized by downstream inflammation, metabolic dysfunction, and cell death manifest from the inflicting primary injury to the head. Secondary injury offers a window for therapeutic interventions, but the multifaceted nature of secondary injury is complicated, necessitating mechanistic tools to screen the efficacy of such interventions. As such, utilizing animal models to define the features of secondary injury mechanisms is critical for medications development. Various animal TBI models employ specialized equipment to recapitulate both primary and secondary injury aspects of human TBI. The organotypic hippocampal slice culture (OHSC) model offers a biological intermediate between live animal and dissociated cell culture models. In OHSC models, *ex vivo* tissue containing heterogenous hippocampal cell types is plated upon permeable culture membranes, which have the capacity to be manipulated. We, therefore, repurposed a commercially available impact device to mechanically distend the OHSC culture membrane, effectively inducing an indirect stretch injury to hippocampal tissue. This stretch injury technique causes characteristic secondary injury trauma, such as widespread cell death, loss of neuronal viability, and production of reactive oxygen species, following the initial insult. Importantly, both the impact force and dwell time of the membrane distention are scalable, a modular feature widely employed across other animal TBI models. This OHSC TBI model may lend itself to high-throughput preliminary assessment of therapeutic efficacy for treatment of secondary injury in animal TBI models.

## Introduction

Traumatic brain injury (TBI) continues to be a prevalent global health issue.^1^ The initial TBI impact causes primary injuries such as skull fracturing, contusions, and axonal shearing^2–5^ that trigger a cascade of excitotoxicity,^6^ mitochondrial dysfunction,^7^ and secondary injuries.^8–9^ While secondary injury provide a therapeutic window to treat TBI,^3,9^ they are complex and complicate TBI treatment.^9–12^ Common approaches to *in vitro* and live animal TBI models include weight drop, controlled cortical impact, and fluid percussion, all of which can produce a variety of injuries in living animals (for review see^13^) *in vitro,* organotypic slice cultures maintain cellular structure, which serves as an important biological intermediate between dissociated cell cultures and live animal studies^14–15^ for further investigation of multifaceted biochemical processes triggered during secondary injury. In organotypic cultures, compression TBI models mimic live animal weight drop models in that a stylus positioned above the tissue is dropped onto a section of tissue.^15–16^ However, the force of injury and deformation of the brain tissue are difficult to standardize.^15^ Alternative stretch injury models can demonstrate tissue deformation during a TBI^15^ by stretching the membrane-bound tissue via compressed gas^17^ or indenter;^18^ however, such models rely on custom-made, noncommercially available equipment, which severely limits model standardization.^15^ Undoubtedly, establishing a high-throughput, reliable, and standardized TBI model for evaluation of secondary injury mechanisms and medications development would benefit the TBI field.

We therefore used the organotypic hippocampal slice culture (OHSC) model in conjunction with a commercially available mechanical impact apparatus to create a new *in vitro* TBI model. We found that tissue damage scaled to the standardized parameters of the duration and force used for membrane distension. This OHSC system provides a highly controllable TBI model that is widely available, user-friendly, and suitable for high-throughput therapeutic screening across different severities and temporal windows of secondary injury.

## Methods

### Organotypic hippocampal slice culture preparation

This experiment was performed at the University of Kentucky in accordance with University of Kentucky IACUC protocols. Male and female Sprague Dawley rat pups (Inotiv Laboratories; Indianapolis, IN) were humanely euthanized at postnatal day 8 and tissue was harvested using aseptic technique as published.^19–20^ Rat brains were placed into cold dissecting media: 97.09% (v/v) Minimum Essential Media (MEM; Invitrogen; Carlsbad, CA, USA), .024M 4-(2-Hydroxyethyl)-1-piperazineethanesulfonic acid (HEPES; Sigma; St. Louis, MO, USA), 0.97% penicillin/streptomycin (Invitrogen), and 1.94% Amphotericin B solution (Sigma). Hippocampi were extracted and sectioned into 200 μm thick slices using a McIllwain Tissue Chopper (Mickle Laboratory Engineering Co. Ltd.; Gomshall, UK). Tissue was placed into petri dishes filled with culture media: 49.26% dissecting media (detailed above), 22.17% double distilled water, 24.63% Heat Inactivated Horse Serum (HIHS; Sigma), 2.46% Hanks Balanced Salt Solution (HBSS; Invitrogen), 0.49% penicillin/streptomycin (Invitrogen), and 0.99% Amphotericin B solution (Sigma). Slices containing the CA1, CA3, and dentate gyrus (DG) regions were plated onto Millicell biopore membrane inserts (0.4 μm, 30 mm diameter; Millipore; MA, USA). While four hippocampal slices were spatially distributed upon the membrane inserts, the specific hippocampal regions were randomly oriented toward the focal site of impact, as warranted from piloted experiments wherein physical manipulation of the tissue upon the membrane jeopardized tissue viability. Inserts were placed into six-well culture plates (5 mL, 9.5 cm^2^ area; VWR; Radnor, PA, USA) containing 1.5 mL of 37°C culture media per well. Tissue was placed into an incubator (37°C at 5% CO_2_) for 5 days before random assignment to a treatment group.

### Injury mechanism

The commercially available Infinite Horizon (IH) Impactor (Precision Systems & Instrumentation; Lexington, KY, USA) performs consistent pre-defined injuries with accessible software to monitor injury parameters for reproducibility.^21^ Repurposed in our design, the IH device was used to deliver an injury to the hippocampal tissue by delivering a desired impact force and dwell time onto the supporting membrane without direct contact to the hippocampus.

#### Injury procedure

**FIG. 1.**
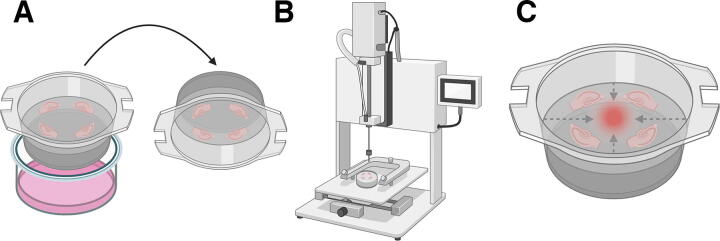
Schematic of injury mechanism using the spinal cord impactor. **(A)** Hippocampal slices were removed from culture media, inverted, and placed onto the spinal cord impactor. **(B)** Culture membrane was then mechanically distended by the spinal cord impactor tip with precise control of force and duration of injury. **(C)** After injury, hippocampal slices were returned to fresh culture media containing 7.48 μM PI or 10 μM DCFH-DA for cell death and reactive oxygen species production, respectively. For neuronal viability endpoints using NeuN immunohistochemistry, slices remained on the injured membrane as in C but were fixed with PFA and incubated in immunohistochemical buffers prior to imaging. For Seahorse assays, slices were removed from the membrane ∼24 h following injury and processed through the Seahorse assay protocol. Note: the hippocampal DG region orientation toward the focal site of impact is for illustrative purposes only; regions were randomly oriented toward the focal site of impact.

At 7 days *in vitro*, tissue was subjected to the injuring procedure (Fig. 1). All tissue was removed from the incubator and taken to the IH device in a surgery suite. For the control group, tissue was brought to the surgery suite for the duration of the injury procedure but was not subjected to injury. For the injury groups, each biopore membrane insert (Millipore) was inverted upon the stage of the IH impactor so that the non-tissue side of the membrane insert was facing the 2.5 mm impactor tip. The rod was positioned 5 mm above the center of the membrane insert, equidistant from the slices. The force (50 kD, 75 kD, 100 kD, or 150 kD) and duration of the dwell time of the impact (0 or 5s) were determined by pilot experiments using IH software (version 5.0.4). After the injury procedure was finished, each membrane insert was placed into new six-well plates filled with fresh, pre-incubated culture media (37°C) that was either plain or contained 7.48 μM propidium iodide (PI) or 10 μM dichloro-dihydro-fluorescein diacetate (DCF) and returned to the incubator.

### Imaging

#### PI staining protocol

Membrane-bound tissue was placed into new six-well culture plates containing 1 mL of 7.48 μM PI (Thermo Fisher; Waltham, MA, USA; #P1304MP; Lots #1493644, #1670391) media. Tissue was imaged 24 h post-injury under a 5x objective lens of a Leica DM-IRB microscope (W. Nuhsbahm Inc.; McHenry, IL, USA) connected to a computer running SPOT advanced software (Windows, version 4.02) via a SPOT 7.2 color mosaic camera (W. Nuhsburg). Images were analyzed using ImageJ software (NIH; Bethesda, MD, USA). An experimenter first manually outlined a background measure and then used an anatomical atlas to manually outline approximate CA1, CA3, and DG subregions of the hippocampus. Readings were converted to a percent control using (S-B)/C, where S is the intensity of the region, B is the background intensity, and C is the mean fluorescent value for the control group’s respective CA1, CA3, and DG intensity for each litter.^22^ This practice was followed for all imaging endpoints. Following PI imaging, tissue upon membrane insert was fixed with 1 mL of 10% formalin solution in culturing plates for 45 min, followed by two 1X phosphate-buffered saline (PBS) washes and placed into new six-well plates of 1 mL of PBS and briefly stored at 4°C for subsequent immunohistochemistry.

#### NeuN staining protocol

After the tissue on the membrane inserts was fixed, the membrane inserts were placed into six-well plates containing buffer: PBS, Triton detergent (1:1000), and 760 nM Bovine Serum Albumin (BSA). 1 mL of buffer was pipetted on top of the tissue and incubated for 45 min at room temperature. Membrane inserts were rinsed twice in PBS and placed into new six-well plates containing 1 mL of PBS. Mouse Anti-NeuN (Millipore-Sigma; #MAB377; Lots #3713321, #3574318; AB_2298772) was diluted 1:200 into the buffer, and 1 mL of this solution was pipetted on top of the inserts. Tissue was incubated at 4°C for 24 h. The following day, inserts were rinsed twice in PBS and placed into new six-well plates containing 1 mL of PBS. Anti-mouse fluorescent secondary antibody conjugated to FITC (Millipore-Sigma; #F8771; Batches #0000119707, #0000132467) was diluted 1:200 in buffer, and 1 mL of this solution was pipetted on top of the membrane inserts. Tissue was returned to 4°C for 24 h. Membrane inserts were washed twice in PBS and placed into new six-well plates containing 1 mL of PBS to be immediately imaged.

#### DCF staining protocol

The procedure for DCFH-DA staining in OHSC was modeled after the protocol by Jung et al.^23^ Briefly, reactive oxygen species (ROS) accumulation in the cultures was assessed 30–60 min post-injury. DCFH-DA (Millipore-Sigma; #D6883-50MG; Lot #059M4133V) was added to fresh culture media at a final 10 μM concentration. Tissue was incubated for 30 min at 37°C. Inserts were then washed twice in PBS and placed into new six-well plates containing 1 mL of PBS to be immediately imaged.

#### Seahorse XFe24 mitochondrial bioenergetics

A subset of hippocampal tissue cultures in the 100 kD-5s injury group were selected 24 h after injury to determine the magnitude of mitochondrial respiration disturbed by an injury that was sufficient to: increase PI update, diminish NeuN fluorescence, and increase DCF fluorescence relative to control across all hippocampal regions. Mitochondria were isolated from 24 tissue slices using the differential centrifugation method and following established mitochondrial bioenergetics protocols previously described.^24^ The Seahorse XFe24 Flux Analyzer (Agilent Technologies; Santa Clara, CA, USA) was employed to measure oxygen consumption rates (OCR) in the presence of various mitochondrial substrates, inhibitors, and uncouplers. Injection ports A to D of the hydrated sensor cartridge were loaded with mitochondrial respiration substrates to measure the OCR in various mitochondrial respiration states, appropriately diluted in respiration buffer without BSA (buffers: 125 mM KCl, ± 0.1% BSA, 20 mM HEPES, 2 mM MgCl2, and 2.5 mM KH2PO4, adjusted to pH 7.2). The final concentrations of the chemicals were as follows: 5 mM pyruvate, 2.5 mM malate, and 2 mM adenosine diphosphate (via Port A; State IIIC1); 2.5 μM oligomycin A (via Port B; State IV); 4 μM carbonyl cyanide-p-trifluoromethoxyphenylhydrazone (FCCP; via Port C; State V-CI); and 1 μM rotenone and 10 mM succinate (via Port D; State V-CII); however, a technical issue precluded analysis of state IV in the Seahorse assay. Isolated mitochondrial proteins were quantified using a Pierce bicinchoninic acid protein assay (Thermo Fisher; #23227). A total of 12 μg of protein was loaded per well in a volume of 50 μL of respiration buffer with BSA. The plates were then centrifuged, and respiration buffer with BSA was gently added to each well of the Seahorse XFe24 plate, resulting in a total volume of 450 μL per well. OCRs were measured based on additions in each injection port on the Seahorse XFe24 Flux Analyzer. Raw OCR values were used for analysis within each experiment. Data were percentage-normalized to the control for each sex and state, and all normalized data are reported in Supplementary Figure S10.

## Results

The full analysis of the relationship between force (50 kD, 75 kD, 100 kD, and 150 kD) and dwell time (0 and 5s) of impact is provided in supplemental figures for PI, NeuN, and DCF in CA1, CA3, and DG regions. For illustrative purposes, we present data from only the highest force (150 kD) at two different dwell times (0 and 5s) compared to the control for each region; additional analyses probing sex effects were performed when appropriate. *t*-Tests, ANOVAs, Fisher’s LSD, and Tukey’s *post hoc* analyses were conducted using SPSS (Version 26; IBM Corporation; Armonk, New York, USA) or GraphPad Prism 10.5.0 (GraphPad Software; Boston, Massachusetts, USA). Figures were generated using Prism 10.5.0 (GraphPad Software) and BioRender.

### Cell death

#### PI in the CA1

**FIG. 2.**
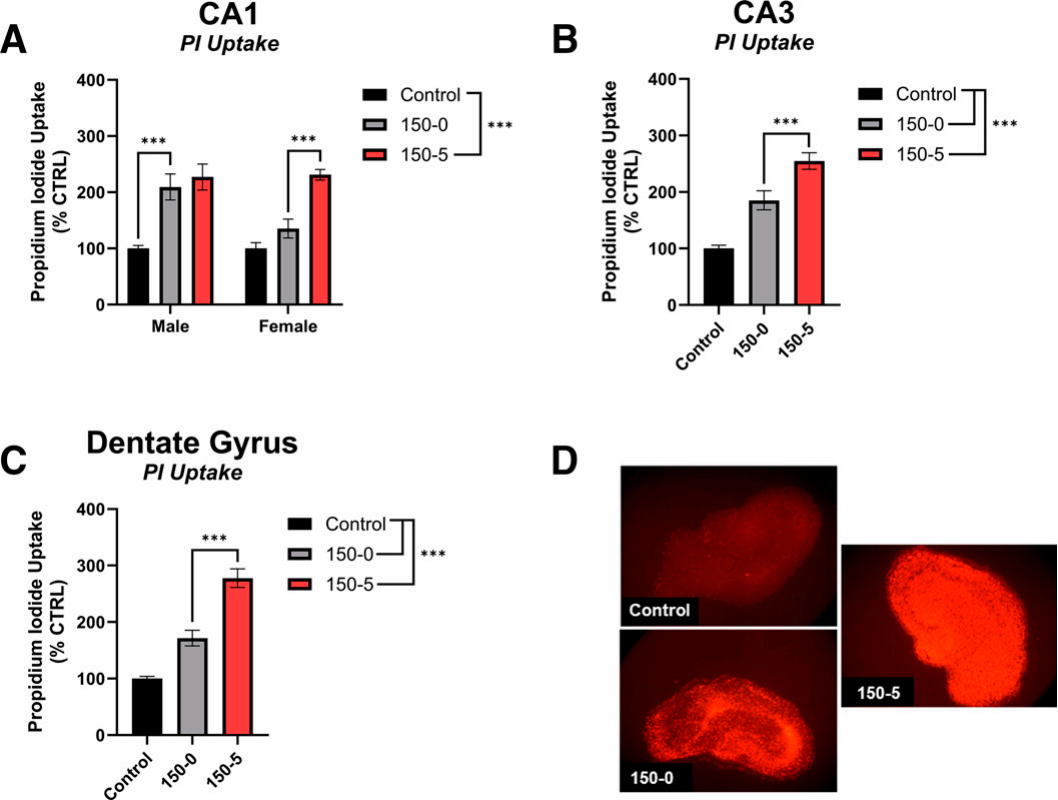
Hippocampal PI Uptake at 150 kD force injury. **(A)** PI in the CA1. The 0s impact at 150 kD exerted more cell death relative to uninjured Control in male tissues. The 5s dwell time at 150 kD exerted more cell death relative to the 0s impact of 150 kD in female tissue. In both male and female tissue, the 5s dwell at 150 kD exerted more cell death relative to the uninjured Control tissue. **(B)** PI in the CA3. Both the 0s and 5s dwell at 150 kD impact exerted more cell death relative to the uninjured Control. The 5s dwell at 150 kD exerted more cell death relative to the 0s dwell time at 150 kD. **(C)** PI in the DG. Both the 0s and 5s dwell at 150 kD impact exerted more cell death relative to the uninjured Control. **(D)** Representative image of PI fluorescence in Control, 150-0s, and 150-5s injury on the organotypic hippocampal slice. *p < 0.05, **p < 0.01, ***p < 0.001.

There was a main effect of sex in the CA1 region of the hippocampus when the injury was analyzed as two separate factors of force and duration, *p* = 0.029, such that the omnibus effect of injury revealed that slices taken from male rats had more PI uptake compared to slices taken from female rats, though this pattern was not consistent across every individual treatment group (Supplementary Fig. S1). A 0s dwell at a 150 kD force increased cell death compared to the control group only in males, *p*-adj = 0.001 (Fig. 2A). Specifically at the 150 kD force, the 5s dwell caused more cell death relative to the 0s dwell only in females, *p*-adj < 0.0001 (Fig. 2A). The 5s dwell time at 150 kD increased PI uptake in the CA1 above Control for both male and female slices, *p*-adj < 0.0001 for both sexes.

#### PI in the CA3

Fisher’s LSD *post hoc* analyses revealed that a 0s dwell time of 150 kD force induced more cell death compared to the control, *p* < 0.001 (Fig. 2B). Further, when the force was applied to the membrane for 5s, PI uptake was significantly higher than the 0s dwell at the same 150 kD force, *p* < 0.001 (Fig. 2B).

#### PI in the DG

Fisher’s LSD *post hoc* analyses revealed that a 0s dwell time of 150 kD force was sufficient to induce increased PI uptake compared to the control, *p* < 0.001 (Fig. 2C). Further, when the force was applied to the membrane for 5s, PI uptake was significantly higher than the 0s dwell at the same 150 kD force, *p* < 0.001 (Fig. 2C). Representative images of PI are found in Figure 2D.

### Neuronal viability

#### NeuN in the CA1

**FIG. 3.**
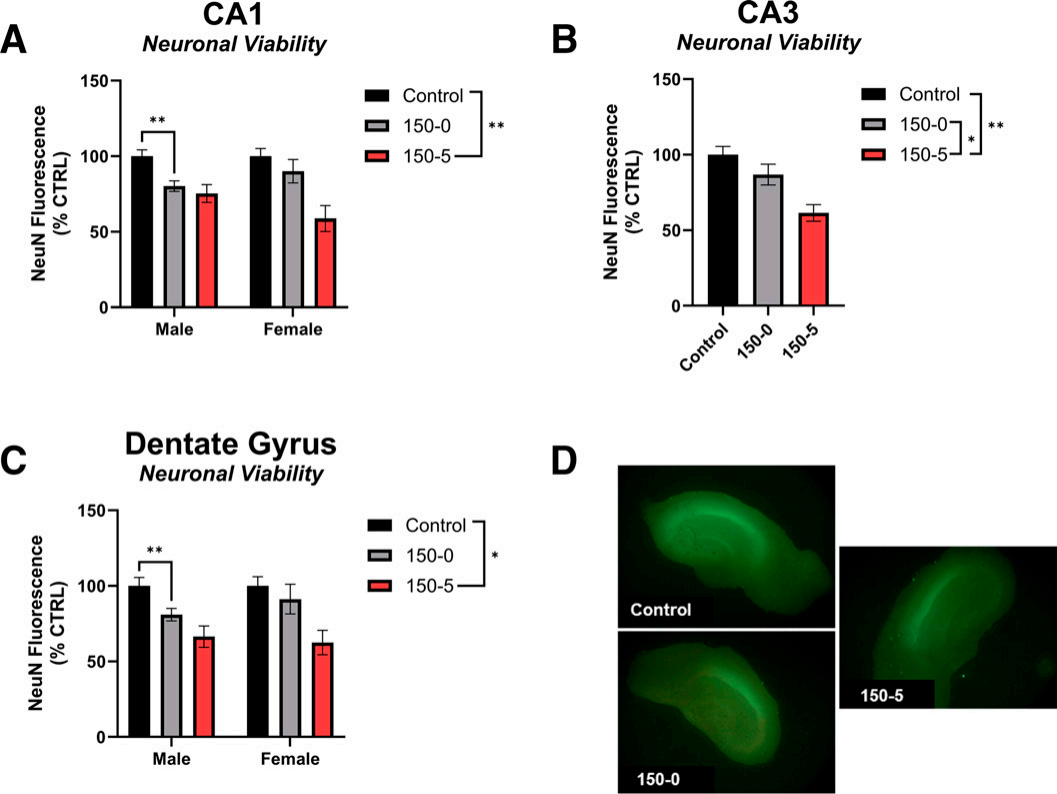
Hippocampal neuronal viability at 150 kD force injury. **(A)** NeuN in the CA1. The 0s impact of 150 kD reduced neuronal viability relative to uninjured Control in male tissue. In both male and female tissue, the 5s dwell at 150 kD reduced neuronal viability relative to the uninjured Control tissue. **(B)** NeuN in the CA3. Both the 0s and 5s dwell at 150 kD impact reduced neuronal viability relative to the uninjured Control. The 5s dwell at 150 kD reduced neuronal viability relative to the 0s dwell time at 150 kD. **(C)** NeuN in the DG. The 0s impact of 150 kD reduced neuronal viability relative to uninjured Control in male tissue. In both male and female tissue, the 5s dwell at 150 kD reduced neuronal viability relative to the uninjured Control tissue. **(D)** Representative image of NeuN fluorescence in Control, 150-0s, and 150-5s injury on the organotypic hippocampal slice. *p < 0.05, **p < 0.01, ***p < 0.001.

There was a main effect of sex in the CA1 region of the hippocampus when the injury was analyzed as two separate factors of force and duration, *p* = 0.022, such that the omnibus effect of injury revealed that slices taken from male rats had less NeuN fluorescence compared to slices taken from female rats, though this pattern was not consistent across every individual treatment group (Supplementary Fig. S4). A 0s dwell at a 150 kD force decreased NeuN fluorescence compared to the control only in males *p*-adj = 0.003 (Fig. 3A). The 5s dwell time at 150 kD reduced NeuN fluorescence below control in the CA1 for both male and female slices, *p*-adj = 0.005 for both sexes.

#### NeuN in the CA3

The 0s dwell at a 150kD force decreased NeuN fluorescence in the CA3; however, when a 5s dwell time was applied, NeuN fluorescence was significantly reduced below control and 0s dwell time levels, *p* < 0.001 and *p* = 0.018, respectively (Fig. 3B).

#### NeuN in the DG

There was a main effect of sex in the DG region of the hippocampus when the injury was analyzed as two separate factors of force and duration, *p* = 0.05, such that the omnibus effect of injury revealed that slices taken from male rats had less NeuN fluorescence compared to slices taken from female rats, though this pattern was not consistent across every individual treatment group (Supplementary Fig. S6). A 0s dwell at a 150 kD force decreased NeuN fluorescence compared to control only in males, *p*-adj = 0.02 (Fig. 3C). The 5s dwell time at 150 kD reduced NeuN fluorescence in the DG below control for both male and female slices, *p*-adj = 0.003 and *p*-adj = 0.02, respectively (Fig. 3C). Representative images of NeuN are found in Figure 3D.

### Reactive oxygen species

#### DCF in the CA1, CA3, and DG

**FIG. 4.**
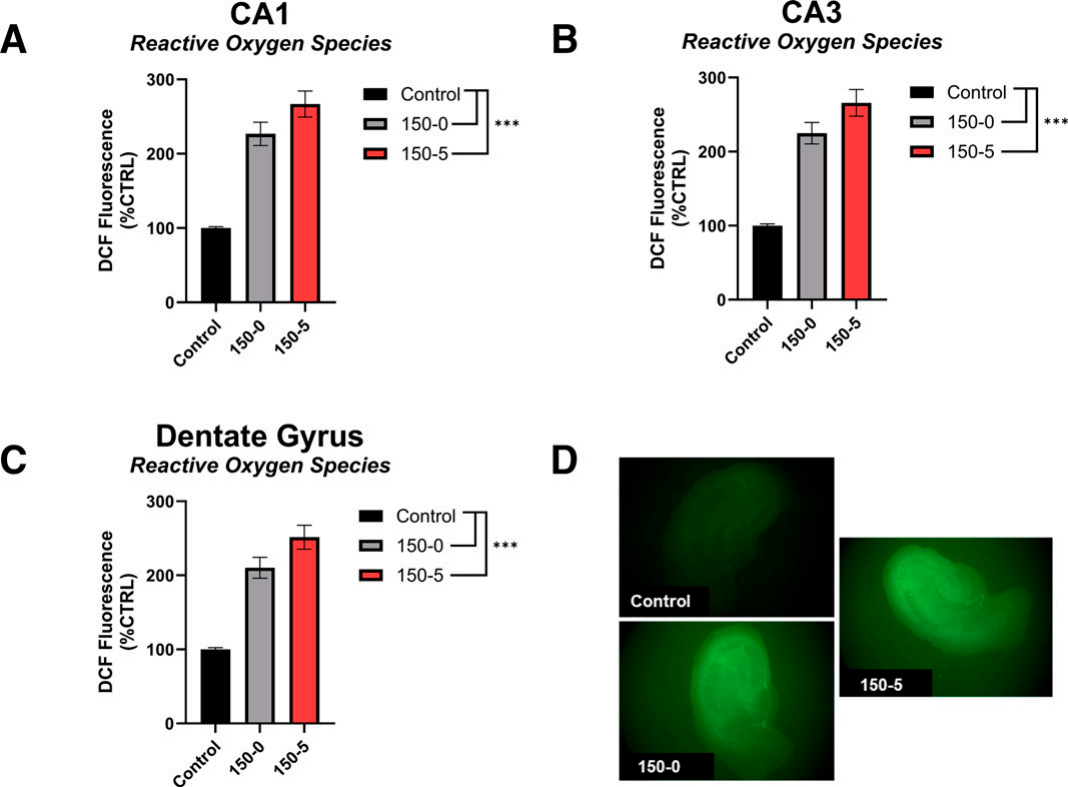
Hippocampal reactive oxygen species production at 150 kD force injury. **(A)** DCF in the CA1. Both the 0s and 5s dwell at 150 kD impact produced more reactive oxygen species relative to uninjured controls. **(B)** DCF in the CA3. Both the 0s and 5s dwell at 150 kD impact produced more reactive oxygen species relative to the uninjured control. **(C)** DCF in the DG. Both the 0s and 5s dwell at 150 kD impact produced more reactive oxygen species relative to the uninjured control. The 5s dwell at 150 kD produced marginally more reactive oxygen species relative to the 0s dwell time at 150kD. **(D)** Representative image of DCF fluorescence in Control, 150-0s, and 150–5 injury on the organotypic hippocampal slice. *p < 0.05, **p < 0.01, ***p < 0.001.

Both the 0s and 5s dwell time at a force of 150 kD increased DCF fluorescence compared to control, *p* < 0.001 in all three CA1, CA3, and DG regions (Fig. 4A–C). Representative images of DCF are found in Figure 4D.

## Discussion

We found that the scale of injury closely mirrored the magnitude of the injury parameters inflicted by distending culture membranes using a commercially available rodent spinal cord injury device.

### Delineation of injury types

The 0s duration caused a significant increase in PI at the 150 kD force for all regions (Fig. 2; Supplementary Figs. S1, S2 and S3). Further, the 0s duration at 100 kD caused a significant increase in PI only in the CA1 and CA3 (Supplementary Figs. S1, S2 and S3). Alternatively, all forces that were held for the duration of 5s caused a significant increase in PI compared to the uninjured control group across all three regions (Supplementary Figs. S1, S2 and S3). Similarly, NeuN immunostaining was also largely unaffected from injury unless the forces were highest (Fig. 3; Supplementary Figs. S4, S5 and S6). Alternatively, DCF demonstrated that ROS production was the most sensitive to our injury parameters (Fig. 4; Supplementary Figs. S7, S8 and S9). Across all three regions, injuries greater than 100 kD-0s resulted in increased DCF fluorescence compared to uninjured control tissue. In the CA1, all injuries greater than 50 kD-5s increased DCF fluorescence compared to uninjured control; however, in the CA3 and DG, the only injury parameter below 100 kD-0s that increased DCF fluorescence was the 75 kD-0s parameter (Supplementary Figs. S8 and S9). While only the 100 kD-5s injury was evaluated for its effects on mitochondrial OCR, this injury parameter was sufficient to decrease State III and State V-CII in both sexes, but only the female mitochondrial oxygen consumption was reduced in State V-CI (Supplementary Fig. S10). Importantly, the 100 kD-5s injury consistently induced injury compared to control slices in all CA1, CA3, and DG regions across all PI, NeuN, and DCF metrics. Taken together, this model provides a highly flexible paradigm that can be manipulated to suit unique research questions and therapeutic strategies targeting secondary injury mechanisms in TBIs.

In the current study, lower injury parameters may have increased non-neuronal cell death, as evidenced by increased PI fluorescence, but did not significantly affect neuronal viability. This injury mechanism was not intended or anticipated to be specific to any one cell type. Though the timing used in this study was optimized for PI, this may have been a suboptimal window for complementary NeuN measures. Future studies should investigate non-neuronal cell types and, more broadly, the role of neuroinflammation in injury endpoints.

### Sex differences

There was a sex effect in the CA1 and DG regions in PI and NeuN fluorescence. Additionally, there were distinctions in mitochondrial bioenergetics between male and female slices in State V-CI of our Seahorse assay. Relatedly, in cortical synaptic mitochondria following a controlled cortical impact injury in mice, bioenergetics in female tissue function properly at 12 h post-injury but become impaired by 24 h post-injury compared to male bioenergetics,^25^ suggesting that the sexually dimorphic bioenergetics findings herein may be an artifact of the 24 h timepoint on which we assayed the injured mitochondria. In any event, the tissue taken in this study was from PND8 rat pups that have not reached sexual maturity.^26–28^ This fact limits our ability to explain susceptibility to cell death, neuronal resilience, or mitochondrial bioenergetics as a function of sex hormones, often reported to contribute to sexually divergent TBI outcomes.^29–31^

### Model considerations and limitations

Injury scaling is a critical aspect in modeling TBI.^13,32,33^ Specifically in this study, distinct impactor tip dwell durations were used as a scaling factor. We observed that the 5s dwell was the most consistent injuring parameter across a variety of forces. While the 5s duration lacks real-world translatability, it fits the purpose of scaling the injury severity, and future studies should explore altering the duration to determine if these results can be obtained with a more realistic duration or if there is a critical threshold for duration to scale up impact injuries. Last, while slice cultures retain some of their structural and physiological organization,^14^ the tissue is disconnected from the body, which may result in cells responding differently than they would in a living animal. Analogous *in vivo* studies would bolster the translational merit of the current model.

## Conclusions

The TBI by mechanical distension model established here can be manipulated to injure at different severities, a feature that mirrors other commonly used TBI models and highlights its candidacy as a promising animal model of TBI in the hippocampus in its own right. Future studies on method optimization, construct validity, and pharmacological intervention to fully characterize this model are necessary and may better inform live animal TBI studies.

## Abbreviations Used

CA1: cornu ammonis 1
CA3: cornu ammonis 3
DCF: dichlorofluorescein
DCFH-DA: dichloro-dihydro-fluorescein diacetate
DG: dentate gyrus
FCCP: carbonyl cyanide-p-trifluoromethoxyphenylhydrazone
IH: Infinite Horizon
kD: kilodyne
OCR: oxygen consumption rate
OHSC: organotypic hippocampal slice culture
PBS: phosphate-buffered saline
PI: propidium iodide
ROS: reactive oxygen species
s: seconds
TBI: traumatic brain injury
