# Supplementary material for: Mechanical Distention of Organotypic Hippocampal Slice Culture Membranes for High-Throughput In Vitro Modeling of Traumatic Brain Injury
Source: Neurotrauma Rep. 2025 Oct 27;6(1):982–90. doi: 10.1177/2689288X251389788 (PMC12670678; doi:10.1177/2689288X251389788)
Supplement: Supplementary Figures [file 2689288x251389788_supplementary_figures.docx]

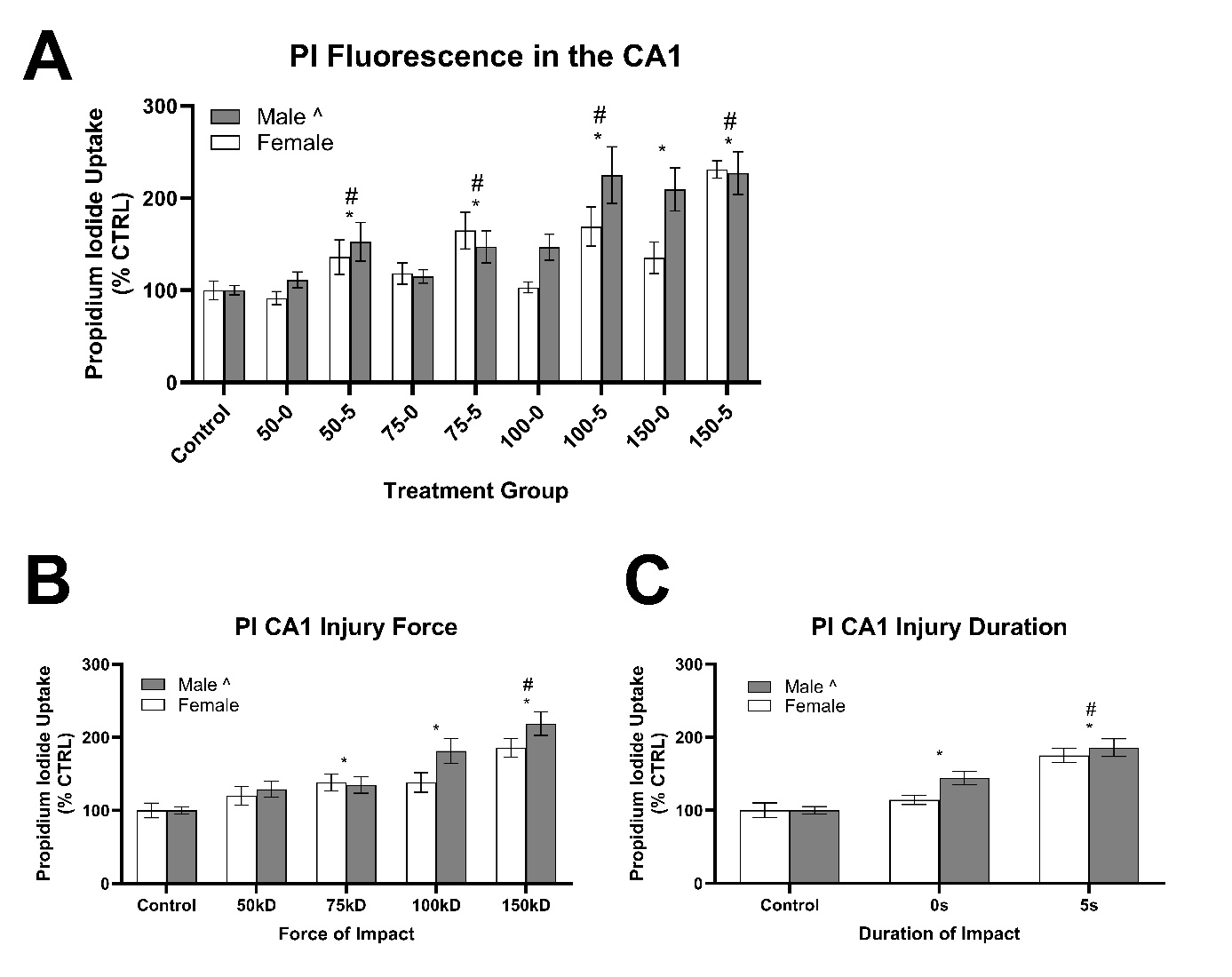
**Supplemental Figure 1.** There was a main effect of sex in the CA1 region of the hippocampus both when the injury is analyzed as a single factor of Treatment, *F*(1, 225) = 6.882, *p* = .009 (Figure 1A) and when the injury is analyzed as two separate factors of Force and Duration *F*(1, 225) = 4.841, *p* = .029 (Figure 1B and 1C). Generally, slices taken from male rats (*M* = 157.5076; *SE* = 7.1487) had significantly more PI uptake compared to slices taken from female rats (*M* = 140.3437; *SE* = 6.0969), though this pattern is not consistent across every treatment group. **(A)** There was a significant main effect of Treatment on propidium iodide uptake, *F*(8, 225) = 14.609, *p* < .001 (Figure 1A). Fisher’s LSD *post hoc* analyses revealed that only the highest force in the 0s duration group (150kD-I) produced a statistically significant increase in PI uptake compared to the Control, (150kD-I *M* = 170.8236; *SE* = 15.8130 vs. Control *M* = 100.00; *SE* = 5.9171), *p* < .001. The lower forces at the 0s duration, 50kD (*M* = 104.2617; *SE* = 6.2308), 75kD (*M* = 117.0523; *SE* = 7.5686), and 100kD (*M* = 126.7481; *SE* = 9.009), did not result in a statistically significant increase in PI uptake compared to the Control group (*M* = 100.00; *SE* = 5.9171), *p* > .05. However, when the force was applied to the membrane for 5ss, every level of force, 50kD (*M* = 143.1862; *SE* =13.7933) *p* = .006, 75kD (*M* = 154.8160; *SE* = 12.9483) *p* < .001, 100kD (*M* = 193.6487; *SE* = 18.4931) *p* < .001, and 150kD (*M* = 229.1267; *SE* = 12.0007) *p* < .001, caused an increase level of PI uptake compared to the Control group (Control *M* = 100.00; *SE* = 5.9171). Fisher’s LSD *post hoc* comparisons were also conducted to determine if the level of injury caused by the extended 5s duration was significantly higher than the 0s duration of the same force. For all forces, 50kD (0s *M* = 104.2617; *SE* = 6.2308 vs. 5s *M* = 143.1862; *SE* = 13.7933), 75kD (0s *M* = 117.0523; *SE* = 7.5686 vs. 5s *M* = 154.8160; *SE* = 12.9483), 100kD (0s *M* = 126.7481; *SE* = 9.009 vs. 5s *M* = 193.6487; *SE* = 18.4931), and 150kD (0s *M* = 170.8236; *SE* = 15.8130 vs. 5s *M* = 229.1267; *SE* = 12.0007), the 5s duration caused a significantly higher uptake of PI compared to the 0s duration of the same force, p = .02, p = .022, p < .001, and p < .001, respectively. **(B)** There was a significant main effect of Force on propidium iodide uptake, *F*(3, 225) =16.818*, p* < .*001* (Figure 1B). Fisher’s LSD *post hoc* analyses revealed that the 75kD (*M* = 136.6335; SE = 8.0009), *p* = .007, 100kD (*M* = 159.5426; *SE* = 11.1143), *p* < .001, and 150kD (*M* = 202.1345; *SE* = 10.4512), *p* < .001, forces caused an increase in PI uptake compared to the Control group (*M* = 100.00; *SE* = 5.9171). However, the lowest force of 50kD (*M* = 124.8256; *SE* = 10.7695) did not differ significantly from the Control group (*M* = 100.00; *SE* = 5.9171), *p* > .05. Fisher’s LSD *post hoc* analyses showed that only the highest force of 150kD was significantly higher than the previous force (150kD *M* = 202.1345; *SE* = 10.4512 vs. 100kD *M* = 159.5426; *SE* = 11.1143), *p* < .001. The 100kD force (M = 159.5426; *SE* = 11.1143) was not significantly higher than the 75kD force (*M* = 136.6335; SE = 8.0009) which was not significantly higher than the 50kD force (*M* = 124.8256; *SE* = 10.7695), p > .05. **(C)** There was a main effect of Duration, *F*(1, 225) =39.463, *p* < .001 in the CA1 region (Figure 1C). Both the 0s (*M* = 129.5681; *SE* = 5.6281) and 5s (*M* = 180.2723; *SE* = 7.7657) durations showed an increased uptake of PI compared to the Control group (*M* = 100.00; *SE* = 5.9171), *p* = .018 and *p* < .001, respectively. The 5s duration also showed a significant increase in PI uptake compared to the 0s duration, *p* < .001. *different from Control; #different from previous injury group.


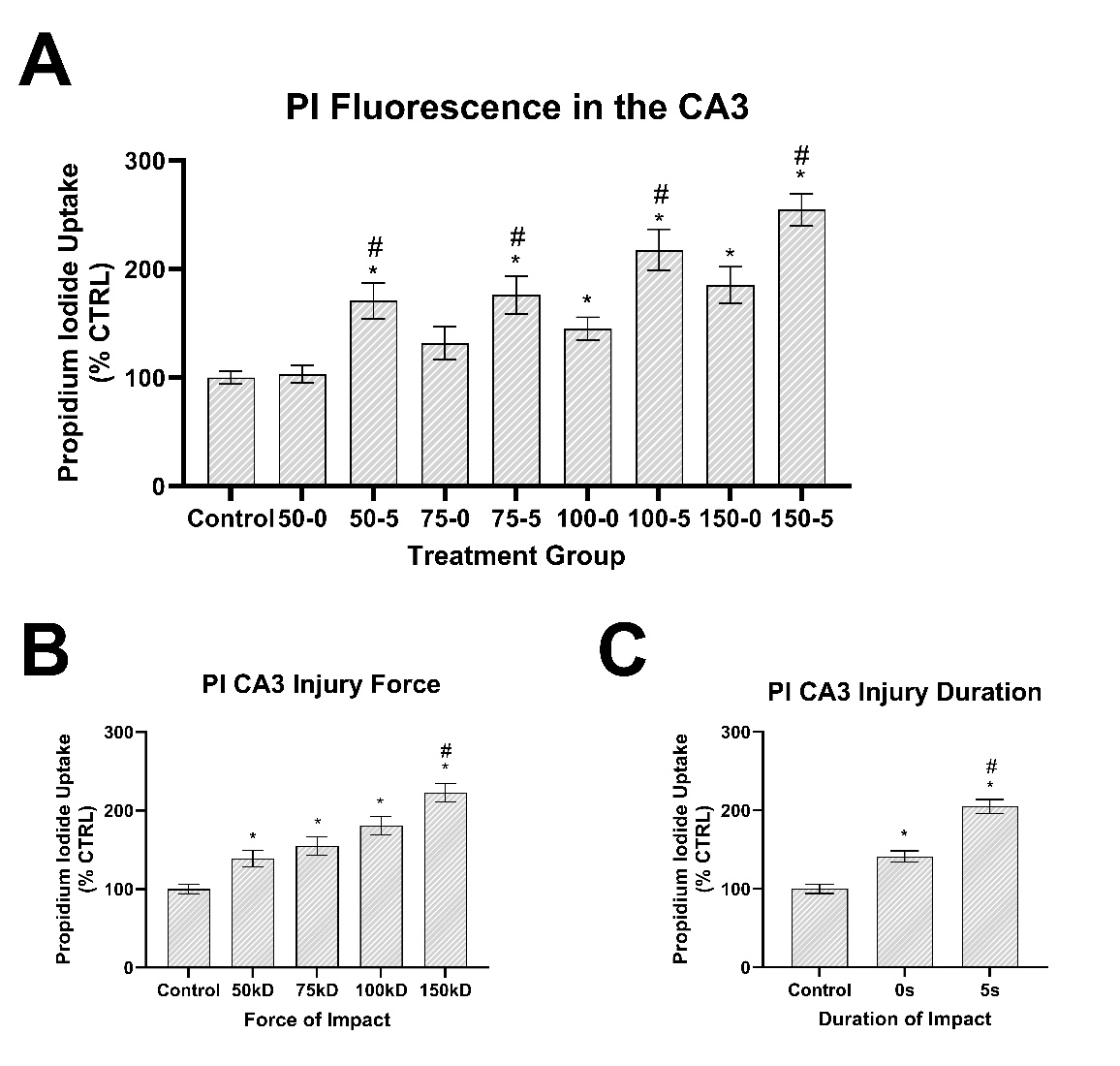
**Supplemental Figure 2.** There was no significant sex effect found in the CA3 region of the hippocampus, so data were collapsed across sex. **(A)** There was a significant main effect of Treatment on propidium iodide uptake in the CA3 region, *F*(8, 234) = 13.215, *p* < .001 (Figure 2A). *Post hoc* analyses using Fisher’s LSD show that the two highest levels of forces with the 0s durations, 100kD (*M* = 145.0723; *SE* = 10.5333) and 150kD (*M* = 185.3076; *SE* = 16.9289), caused an increase in PI uptake compared to the Control group (*M* = 100.00; *SE* = 5.9183), *p* = .023 and *p* < .001, respectively. The lower forces with an 0s duration, 50kD (*M* = 103.1283; *SE* = 8.1211) and 75kD (*M* = 131.7919; *SE* = 15.1046) did not cause a significant increase in PI uptake compared to the Control (*M* = 100.00; *SE* = 5.9183), *p* > .05. However, when the force was applied to the membrane for 5ss, every level of force, 50kD (*M* = 170.7993; *SE* = 16.3922) *p* < .001, 75kD (*M* = 176.2959; *SE* = 17.4343) *p* < .001, 100kD (*M* = 217.6331; *SE* = 19.0484) *p* < .001, and 150kD (*M* = 254.7629; *SE* = 14.7315) *p* < .001, caused an increase level of PI uptake compared to the Control group (Control *M* = 100.00; *SE* = 5.9183). Fisher’s LSD *post hoc* comparisons were also conducted to determine if the level of injury caused by the extended 5s duration was significantly higher than the 0s duration of the same force. For all forces, 50kD (0s *M* = 103.1283; *SE* = 8.1211 vs. 5s *M* = 170.7993; *SE* =16.3922), 75kD (0s *M* = 131.7919; *SE* = 15.1046 vs. 5s *M* =176.2959; *SE* = 17.4343), 100kD (0s *M* = 145.0723; *SE* = 10.5333 vs. 5s *M* = 217.6331; *SE* = 19.0484), and 150kD (0s *M* = 185.3076; *SE* = 16.9289 vs. 5s *M* = 254.7629; *SE* = 14.7315), the 5s duration caused a significantly higher uptake of PI compared to the 0s duration of the same force, p = .001, p = .029, p < .001 and p < .001, respectively. **(B)** There was a significant main effect of Force on propidium iodide uptake in the CA3, *F*(3, 234) = 12.705, *p* < .001 (Figure 2B). All forces, 50kD (*M* = 138.879; *SE* = 10.4887), *p* = .022, 75kD (*M* = 154.8680; *SE* = 11.8927), p = .001, 100kD (*M* = 180.6413; *SE* = 11.8309), *p* < .001, and 150kD (*M* = 222.6077; *SE* = 12.0118), *p* < .001 caused significant increase in PI fluorescence compared to the Control group (*M* = 100.00; *SE* = 5.9183). Fisher’s LSD *post hoc* analyses showed that only the highest force of 150kD was significantly higher than the previous force (150kD *M* = 222.6077; *SE* = 12.0118 vs. 100kD *M* = 180.6413; *SE* = 11.8309), *p* = .004. The 100kD force (*M* = 180.6413; *SE* = 11.8309) was not significantly higher than the 75kD force (*M* = 154.8680; *SE* = 11.8927) which was not significantly higher than the 50kD force (*M* = 138.879; *SE* = 10.4887), *p* > .05. **(C)** There was a main effect of Duration, *F*(1, 234) = 38.662, *p* < .001 (Figure 2C). Both the 0s (*M* = 141.2683; *SE* = 7.1012) and 5s (*M* = 204.9783; *SE* = 8.9409) durations showed an increased uptake of PI compared to the Control group (*M* = 100.00; *SE* = 5.9183), *p* = .007 and *p* < .001, respectively. The 5s duration also showed a significant increase in PI uptake compared to the 0s duration, *p* < .001. *different from Control; #different from previous injury group.


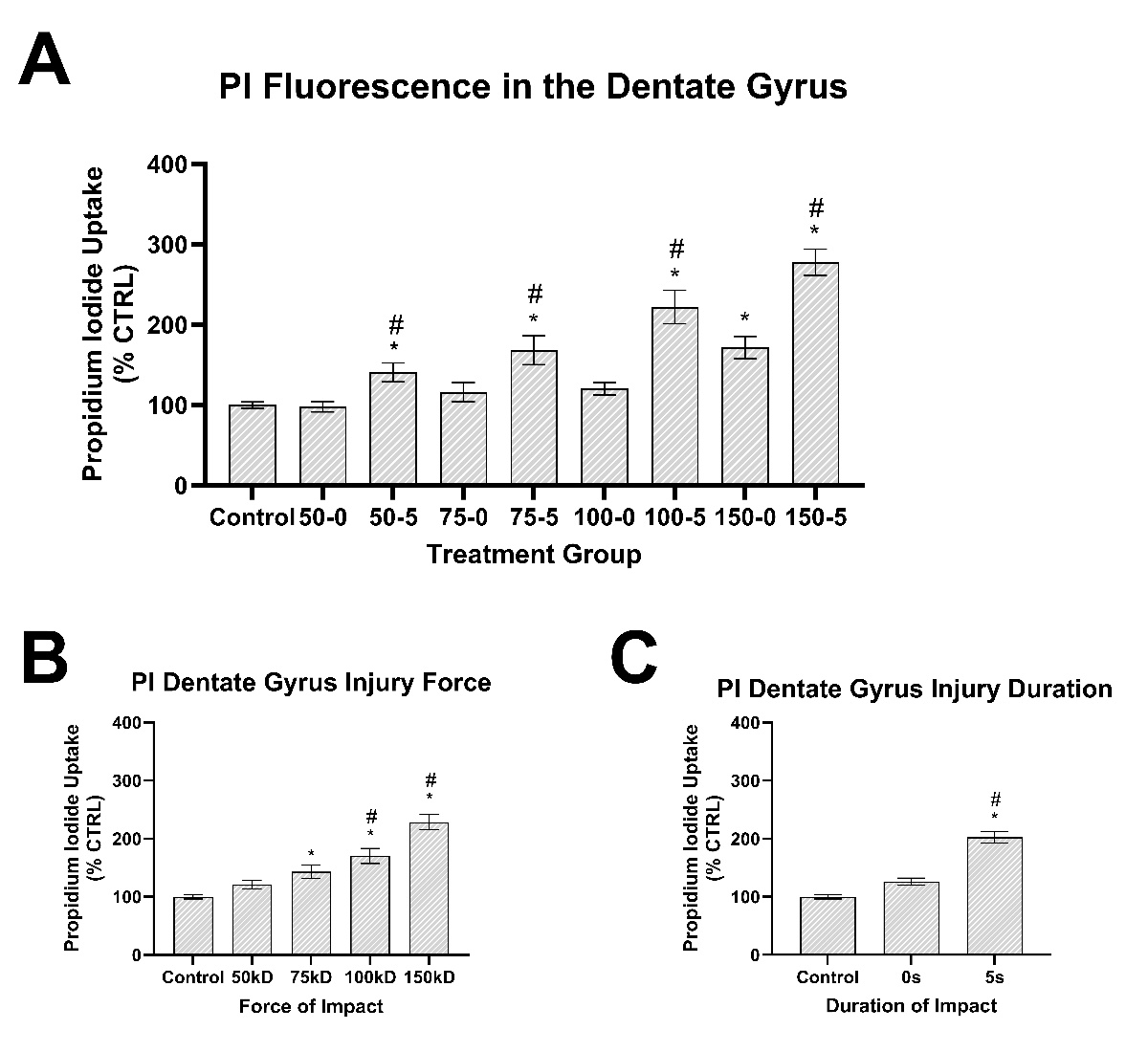
**Supplemental Figure 3.** There was no significant sex effect found in the DG region of the hippocampus, so data were collapsed across sex. **(A)** There was a significant main effect of Treatment on propidium iodide uptake in the DG region of the hippocampus, *F*(8, 234) = 21.105, *p* < .001 (Figure 3A). *Post hoc* analyses (Fisher’s LSD) revealed that only the highest force in the 0s duration group (150kD-I) produced a statistically significant increase in PI uptake compared to the Control, (150kD-I *M* = 171.663; *SE* = 13.9325 vs. Control *M* = 100.00; *SE* = 3.8359), *p* < .001. The lower forces at the 0s duration, 50kD (*M* = 97.9449; *SE* = 6.3982), 75kD (*M* = 116.0942; *SE* = 12.0120), and 100kD (*M* = 120.3978; *SE* = 7.8675), did not result in a statistically significant increase in PI uptake compared to the Control group (*M* = 100.00; *SE* = 3.8359), *p* > .05. At the longer 5s duration, all forces, 50kD (*M* 140.7057; *SE* = 11.7339), *p* = .025, 75kD (*M* = 168.3015; *SE* = 18.0572), p < .001, 100kD (*M* = 222.1465; *SE* = 20.8163), *p* < .001 and 150kD (*M* = 277.6521; *SE* = 16.6250), *p* < .001 caused increased PI fluorescence compared to the Control group (*M* = 100.00; *SE* = 3.8359). Fisher’s LSD *post hoc* comparisons were also conducted to determine if the level of injury caused by the extended 5s duration was significantly higher than the 0s duration of the same force. For all forces, 50kD (0s *M* = 97.9449; *SE* = 6.3982 vs. 5s *M* = 140.7057; *SE* = 11.7339), 75kD (0s *M* = 116.0942; *SE* = 12.0120 vs. 5s *M* =168.3015; *SE* = 18.0572), 100kD (0s *M* = 120.3978; *SE* = 7.8675 vs. 5s *M* =222.1465; *SE* = 20.8163), and 150kD (0s *M* = 171.6630; *SE* = 13.9325 vs. 5s *M* =277.6521; *SE* = 16.6250), the 5s duration caused a significantly higher uptake of PI compared to the 0s duration of the same force, p = .026, p = .006, p < .001, and p < .001, respectively. **(B)** There was a significant main effect of Force on propidium iodide uptake, *F*(3, 234) = 22.997, *p* < .001 (Figure 3B). Fisher’s LSD *post hoc* analyses revealed that the 75kD (*M* = 143.1647; SE = 11.3417), *p* = .006, 100kD (*M* = 170.2746; *SE* = 13.0185), *p* < .001, and 150kD (*M* = 228.5830; *SE* = 13.1077), *p* < .001, forces caused an increase in PI uptake compared to the Control group (*M* = 100.00; *SE* = 3.8359). However, the lowest force of 50kD (*M* = 120.5355; *SE* = 7.4460) did not differ significantly from the Control group (*M* = 100.00; *SE* = 3.8359), *p* > .05. Fisher’s LSD *post hoc* analyses showed that only the highest forces of 150kD and 100kD were significantly higher than the previous forces (150kD *M* = 228.5830; *SE* = 13.1077 vs. 100kD *M* = 170.2746; *SE* = 13.0185), *p* < .001. The 100kD force (*M* = 170.2746; *SE* = 13.0185) was significantly higher than the 75kD force (*M* = 143.1647; *SE* = 11.3417). However, the 75kD force was not significantly higher than the 50kD force (*M* = 120.5355; *SE* = 7.4460), *p* > .05. **(C)** There was a main effect of Duration, *F(*1, 234) = 63.006, *p* < .001 (Figure 3C). The 5s duration (M = 202.3434; SE = 9.7855) caused a significant increase in PI fluorescence compared to the Control group (M = 100.00; SE = 3.859). However, the 0s duration group (*M* = 126.3626; *SE* = 5.8305) did not cause a significant increase in PI uptake versus the Control group. The 5s duration also showed a significant increase in PI uptake compared to the 0s duration, *p* < .001. *different from Control; #different from previous injury group.


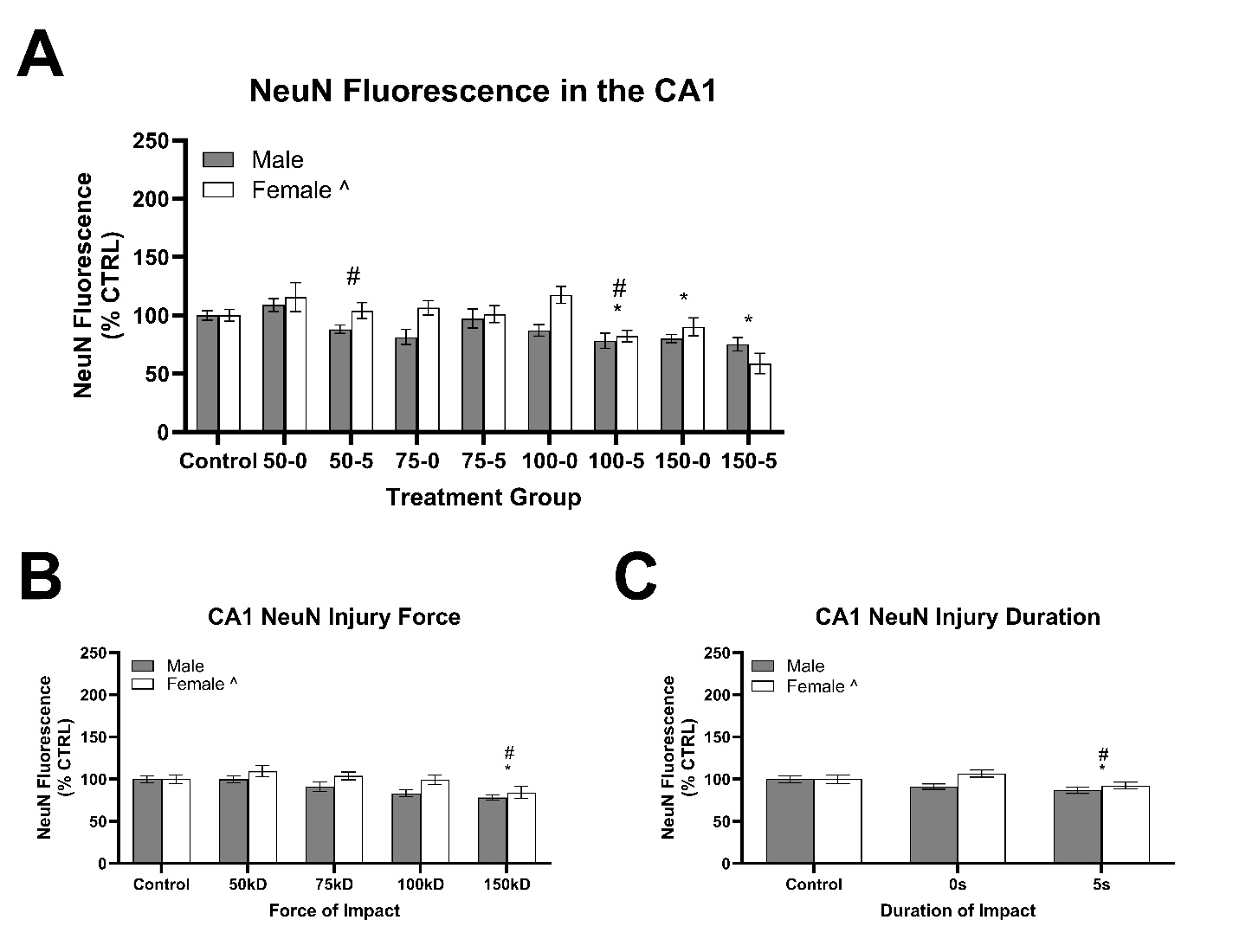
**Supplemental Figure 4.** There was a main effect of sex in the CA1 region of the hippocampus both when the injury is analyzed as a single factor of Treatment, *F*(8, 198) = 7.136, *p* = .008 (Figure 4A) and when the injury is analyzed as two separate factors of Force and Duration *F*(1, 198) = 5.356, *p* = .022 (Figure 4B and 4C). Generally, slices taken from male rats (*M* = 90.3739; *SE* = 2.1433) had a greater reduction in NeuN compared to slices taken from female rats (*M* = 100.0974; *SE* = 2.6352). **(A)** There was a significant effect of Treatment in the CA1 region of the hippocampus, *F*(8, 198) = 5.884, *p* < .001 (Figure 4A). Post hoc analyses (Fisher’s LSD) indicate that the highest 0s force, 150kD-I (*M* = 85.3182; *SE* = 4.3699), *p* = .016, and the two highest forces of long duration, 100kD-5 (*M* = 80.4206; *SE* = 3.9140), *p* = .002 and 150kD-5 (*M* = 70.7768; *SE* = 5.2097), *p* < .001, resulted in a significant reduction in NeuN immunofluorescence compared to the Control group (*M* = 100.00; *SE* = 3.2732). The other forces with an 0s duration, 50kD (*M* = 111.2861; *SE* = 5.5561), 75kD (*M* = 95.6307; *SE* = 5.1284), and 100kD (*M* = 101.1516; *SE* = 5.1739) did not cause a significant decrease in NeuN compared to the Control group (*M* = 100.00; *SE* = 3.2732), *p* > .05. Additionally, for the prolonged duration, the lower forces, 50kD (*M* = 95.7392; *SE* = 4.0981) and 75kD (*M* = 98.8244; *SE* = 5.5009), also did not result in a significant decrease of NeuN immunofluorescence compared to Control, *p* > .05. Additional *post hoc* comparisons (Fisher’s LSD) also determined if the level of injury caused by the extended 5s duration was significantly higher than the 0s duration of the same force. For the 50kD force, there was a significant decrease in NeuN immunofluorescence in the longer 5s duration (*M* = 95.7392; *SE* = 4.0981) compared to the 0s duration (*M* = 111.2861; *SE* = 5.5561), *p* = .018. There was also a significant decrease in the NeuN immunofluorescence from the 5s duration (*M* = 80.4206; *SE* = 3.9140) compared to the 0s duration, (*M* = 101.1516; *SE* = 5.1739), *p* = .001. However, for the 75kD (0s *M* = 95.6307; *SE* = 5.1284 vs 5s *M* = 98.8244; *SE* = 5.5009) and the 150kD (0s *M* = 85.3182; *SE* = 4.3699 vs 5s *M* = 70.7768; *SE* = 5.2097) groups, there was no significant difference between the two durations, *p* > .05. **(B)** There was a significant main effect of Force on NeuN immunofluorescence, *F*(3, 225) =16.818*, p* < .*001* (Figure 4B). Fisher’s LSD *post hoc* analyses revealed that only the highest force of 150kD (*M* = 80.8750; *SE* = 3.5700) resulted in significant reduction in NeuN signal compared to the Control (*M* = 100.00; *SE* = 3.2733) group, *p* < .001. The lower forces 50kD (*M* = 103.8366; *SE* = 3.6415), 75kD (*M* = 97.3841; *SE* = 3.7737), and 100kD (*M* = 91.2007; *SE* = 3.5697) did not cause a significant reduction in NeuN immunofluorescence compared to Control (*M* = 100.00; S*E* = 3.2733), *p* > .05. Similarly, the only force that cause significantly more reduction in NeuN compared to the previous force was the 150kD group (*M* = 80.8750; *SE* = 3.5700) compared to the 100kD group (*M* = 91.2007; *SE* = 3.5697), *p* = .038. **(C)** There was a significant effect of Duration on NeuN immunofluorescence in the CA1, *F*(1, 198) = 12.788, *p* < .001. The 0s duration (*M* = 98.4298; *SE* = 2.6763) did not differ from the Control group (*M* = 100.00; *SE* = 3.2733), *p* > .05. The five second duration (*M* = 89.2759; *SE* = 2.6665) resulted in a significant decrease in NeuN compared to the Control (*M* = 100.00; *SE* = 3.2733), *p* = .024 and the 0s duration group (*M* = 98.4298; *SE* = 2.6763), *p* = .006. *different from Control; #different from previous injury group.


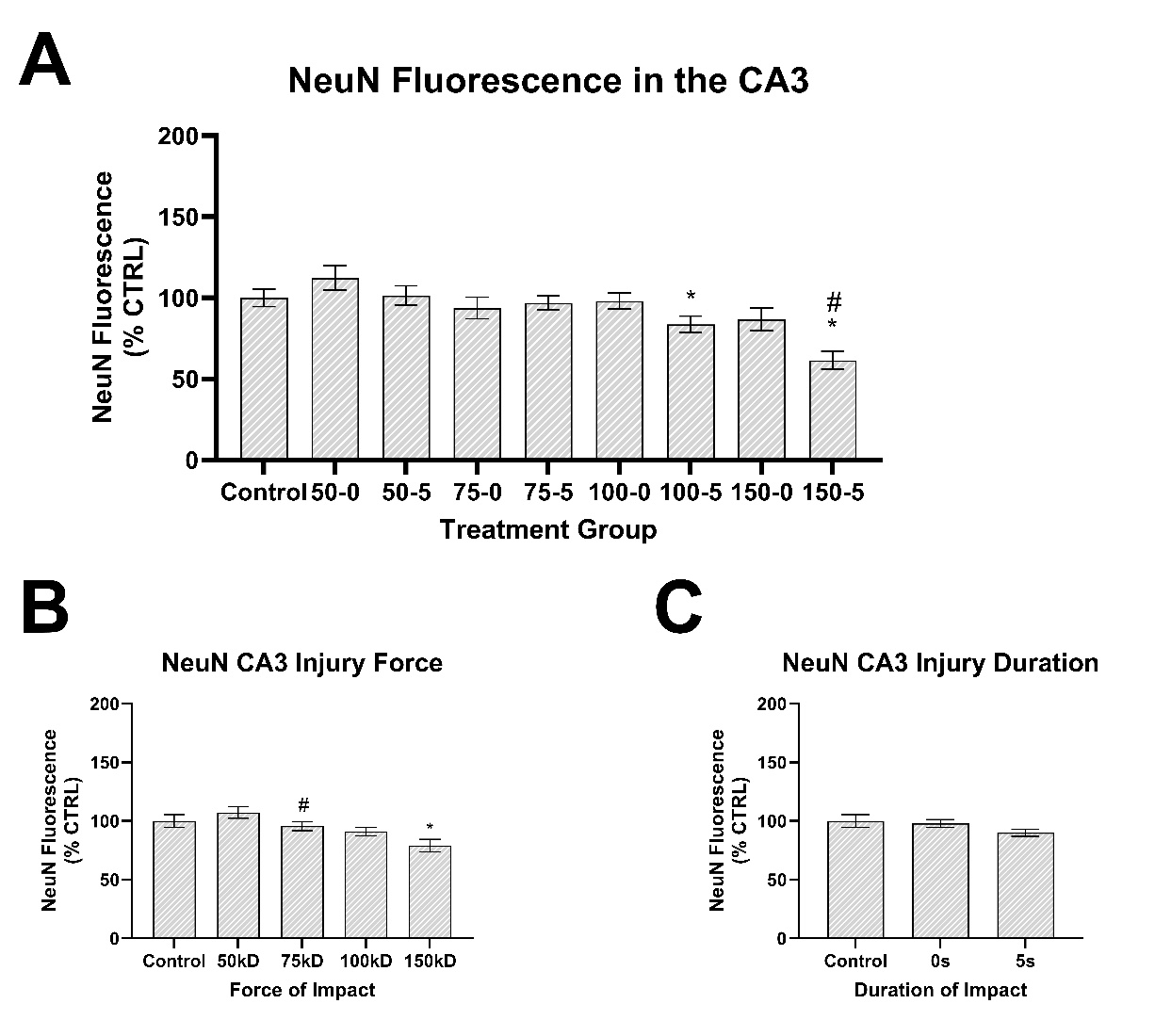
**Supplemental Figure 5.** There was no significant sex effect found in the CA3 region of the hippocampus, so data were collapsed across sex. **(A)** There was a significant effect of Treatment in the CA3 region of the hippocampus, *F*(8, 207) = 3.909, *p* < .001 (Figure 5A). The two highest forces in the 5s duration, 100kD (*M* = 83.6397; *SE* = 5.0579) and 150kD (*M* = 61.4226; *SE* = 5.4903), caused a significant decrease in NeuN compared to the Control, *p* = .041 and *p* < .001, respectively. The other forces at the 5s duration, 50kD (*M* = 101.4384; *SE* = 5.9051) and 75kD (*M* = 96.9793; *SE* = 4.2393), did not cause a reduction in NeuN compared to the Control (*M* = 100.00; *SE* = 5.4830), p > .05. Similarly, none of the forces at the 0s duration, 50kD (*M* = 112.4075; *SE* = 7.5495), 75kD (*M* = 93.7977; *SE* = 6.6420), 100kD (*M* = 98.0545; *SE* = 5.0120), and 150kD (*M* = 86.8188; *SE* = 6.8694), had an effect on NeuN fluorescence, *p* > .05. Fisher’s LSD *post hoc* comparisons were also conducted to determine if the level of injury caused by the extended 5s duration was significantly higher than the 0s duration of the same force. Only the highest force level of 150kD showed a difference between the 0s and 5s durations (0s *M* = 86.8188; *SE* = 6.8694 vs 5s *M* = 61.4226; *SE* = 5.4903), *p* = .018. The other forces, 50kD (0s *M* = 112.4075; *SE* = 7.5495 vs 5s *M* = 101.4284; *SE* = 5.9051), 75kD (0s *M* = 93.7977; *SE* = 6.64202 vs 5s *M* = 96.9793; *SE* = 4.2393), and 100kD (0s *M* = 98.0545; *SE* = 5.0120 vs 5s *M* = 83.6397; *SE* =5.0579), did not have significant differences between the two durations, *p* > .05. **(B)** There was a significant main effect of Force on NeuN immunofluorescence in the CA3, *F*(3, 207) = 8.008, *p* < .001 (Figure 5B). The 150kD force (*M* = 79.0589, *SE* = 5.3863) resulted in a decrease of NeuN fluorescence compared to the Control (*M* = 100.00; *SE* = 5.4830), *p* = .004. However, the lower forces, 50kD (*M* = 107.1467; *SE* = 4.8600), 75kD (*M* = 95.5445; *SE* = 3.7593), and 100kD (*M* = 91.1354; *SE* = 3.6724), did not show a significant reduction in NeuN immunofluorescence versus the Control (*M* = 100.00; *SE* = 5.4830), *p* > .05. Although not significantly reduced from the level of NeuN immunofluorescence seen in Control, the 75kD group (*M* = 95.5445; *SE* = 3.7593), was significantly lower than the previous force of 50kD (*M* = 107.1467; *SE* = 4.8600), p = .05. None of the other forces showed decreased NeuN fluorescence compared to the previous force, 100kD vs 75kD (100kD *M* = 91.1354; *SE* = 3.6724 vs. 75kD *M* = 95.5445; *SE* = 3.7593) and 150kD vs 100kD (*M* = 79.0589, *SE* = 5.3863 vs. 100kD *M* = 91.1354; *SE* = 3.6724), p > .05. **(C)** There was a significant main effect of Duration on NeuN immunofluorescence in the CA3, *F*(1, 207) = 7.062, *p* = .008 (Figure 5C). However, none of the pairwise comparisons reached the level of significance needed. The 0s duration (*M* = 97.8528; *SE* = 3.3653) and the 5s duration (*M* = 89.8986; *SE* = 2.9355) did not differ from the Control group (*M* = 100.00; *SE* = 5.4830), *p* = .722 and *p* = .101, respectively. The 5s duration (*M* = 89.8986; *SE* = 2.9355) also failed to reach significance from the 0s duration (*M* = 97.8528; *SE* = 3.3653), *p* = .067. *different from Control; #different from previous injury group.


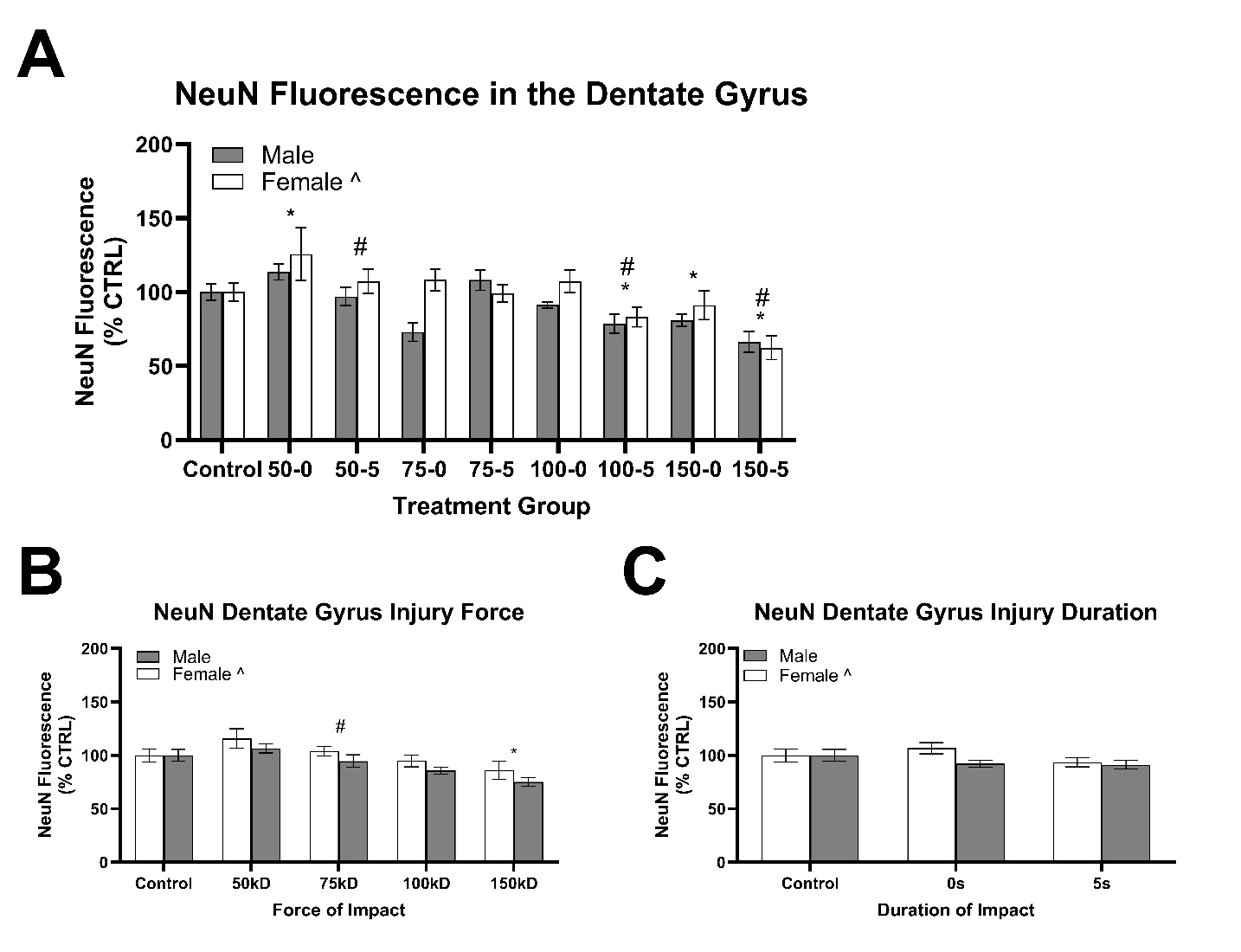
**Supplemental Figure 6.** There was a main effect of sex in the DG region of the hippocampus both when the injury is analyzed as a single factor of Treatment, *F*(1, 198) = 5.184, *p* = .024 (Figure 6A) and when the injury is analyzed as two separate factors of Force and Duration *F*(1, 198) = 3.891, *p* = .05 (Figure 6B and 6C). Generally, slices taken from male rats (*M* = 92.8454; *SE* = 2.3228) had a greater reduction in NeuN compared to slices taken from female rats (*M* = 100.5491; *SE* = 3.0818), though there is some variation between groups. **(A)** There was a significant main effect of Treatment in the DG region of the hippocampus, *F*(8, 198) = 6.571, *p* < .001 (Figure 6A). Fisher’s LSD indicated that the highest 0s force, 150kD-I (*M* = 86.2888; *SE* = 5.4578), *p* = .044, and the two highest forces of long duration, 100kD-5 (*M* = 81.1070; *SE* = 4.5569), *p* = .006 and 150kD-5 (*M* = 65.3418; *SE* = 5.4085), *p* < .001, resulted in a significant reduction in NeuN immunofluorescence compared to the Control group (*M* = 100.00; *SE* = 4.0864). Interestingly, the 50kD-I treatment group (*M* = 118.0423; *SE* = 7.1490) had significantly higher NeuN compared to the Control group (*M* = 100.00; *SE* = 4.0864), *p* = .008. The remaining forces with 0s duration, 75kD (*M* = 92.9825; *SE* = 6.1061) and 100kD (*M* = 98.7581; *SE* = 3.9398), failed to differ significantly from the Control group, *p* > .05. The lower forces with the 5s duration, 50kD (*M* = 102.0503; *SE* = 5.0947) and 75kD (*M* = 104.3221; *SE* = 4.6906) did not differ significantly from the Control group (*M* = 100.00; *SE* = 4.0864), *p* > .005. Fisher’s LSD *post hoc* comparisons were also conducted to determine if the level of injury caused by the extended 5s duration was significantly higher than the 0s duration of the same force. For 50kD, there was a significant reduction in NeuN fluorescence in the 5s duration (*M* = 102.0503; *SE* = 5.0947) compared to the 0s duration (*M* = 118.0423; *SE* = 7.1490), *p* = .029. This was also true of the 100kD (5s *M* = 81.1070; *SE* = 4.5569 vs. 0s *M* = 98.7581; *SE* = 3.9398), *p* = .014 and 150kD (5s *M* = 65.3418; *SE* = 5.4085 vs. 0s *M* = 86.2888; *SE* = 5.4578) forces, *p* = .023, but the 75kD force durations did not significantly differ from each other (5s *M* = 104.3221; *SE* = 4.6906 vs. 0s *M* =92.9825; *SE* = 6.1061), *p* > .05. **(B)** There was a significant main effect of Force on NeuN immunofluorescence in the DG, *F*(3, 198) = 12.164, *p* < .001 (Figure 6B). Fisher’s LSD *post hoc* analyses revealed that the highest force of 150kD (*M* = 79.8883; *SE* = 4.4044) caused a significant decrease in NeuN compared to the Control (*M* = 100.00; *SE* = 4.0864), *p* = .001. The lower forces of 50kD (*M* = 110.3795; *SE* = 4.5577), 75kD(*M* = 99.2082; *SE* = 3.8155), and 100kD (*M* = 90.2855; *SE* = 3.2224) were not significantly different from Control (*M* = 100.00; *SE* = 4.0864), *p* > .05. Fisher’s LSD *post hoc* comparisons were also conducted to determine if the level of force resulted in significantly less NeuN fluorescence than the previous force. Compared to the 50kD (M = 110.3795; SE = 4.5577), the 75kD group (M = 99.2082; SE = 3.8155) showed less NeuN immunofluorescence, p = .029. However, there was no significant difference between the 75kD (M = 99.2082; SE = 3.8155) and 100kD group (M = 90.2855; SE = 3.2224), nor the 100kD (M = 90.2855; SE = 3.2224) and the 150kD group (M = 79.8883; SE = 4.4044), p > .05. **(C)** There was a significant main effect of Duration on NeuN immunofluorescence in the DG, *F*(1, 198) = 7.743, *p* = .006 (Figure 6C). However, none of the pairwise comparisons reached the level of significance needed. The 0s duration (*M* = 99.1372; *SE* = 3.0698) and the 5s duration (*M* = 92.250; *SE* = 2.9048) did not differ from the Control group (*M* = 100.00; *SE* = 4.0864), *p* = .868 and *p* = .144, respectively. The 5s duration (*M* = 92.250; *SE* = 2.9048) also failed to reach significance from the 0s duration (*M* = 99.1372; *SE* = 3.0698), *p* = .065. *different from Control; #different from previous injury group.


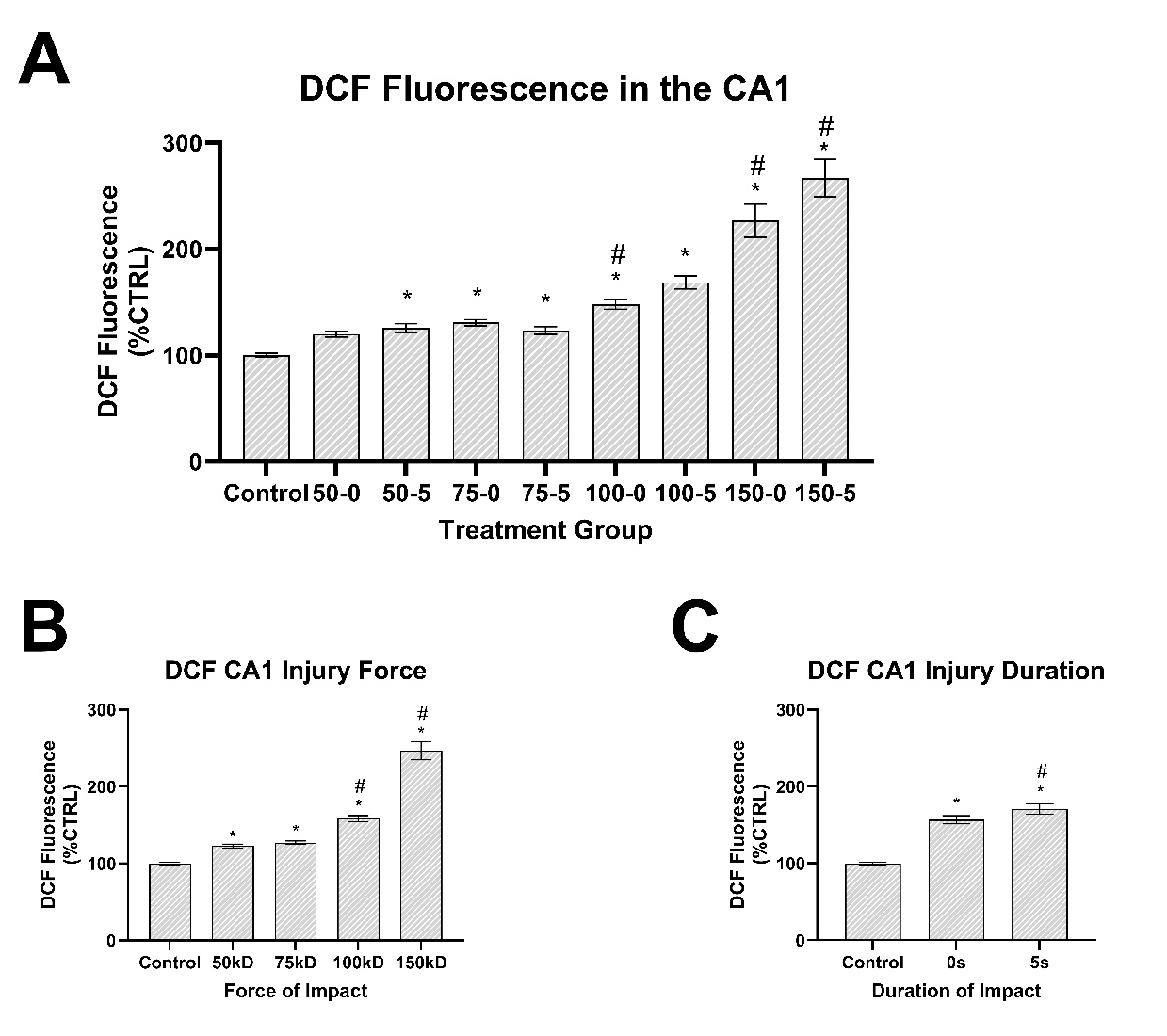
**Supplemental Figure 7.** There was no significant sex effect found in the CA1 region of the hippocampus, so data were collapsed across sex. **(A)** There was a main effect of Treatment in the CA1 region of the hippocampus, *F*(8, 348) = 42.082, *p* < .001 (Figure 7A). Fisher’s LSD *post hoc* analyses revealed that all levels of force held for a 5s duration, 50kD (*M* = 125.7108; *SE* = 4.1981), *p* = .033, 75kD (*M* = 123.5677; *SE* = 3.6294), *p* = .05, 100kD (*M* = 168.8455; *SE* = 6.1463), *p* < .001, and 150kD (*M* = 266.9611; *SE* = 17.4814), *p* < .001, resulted in increased DCF fluorescence compared to the uninjured Control group (*M* = 100.00; *SE* = 1.9495). Additionally, the three highest forces with an 0s also caused an increase in DCF fluorescence, 75kD (*M* = 130.7717; *SE* = 2.8324), *p* = .011, 100kD (*M* = 147.9892; *SE* = 4.6903), *p* < .001, and 150kD (*M* = 226.8258; *SE* = 15.5943), *p* < .001. The only injury treatment that did not cause a significant increase in DCF was the lowest 50kD-I group (*M* = 120.0173; *SE* = 2.5797), *p* > .05. Post hoc analyses also tested if the level of injury caused significantly more than the previous injury level. The 50kD-5 (*M* = 125.7108; *SE* = 4.1981) did not differ from the 50kD-I (*M* = 120.0173; *SE* = 2.5797), the 75kD-I (*M* = 130.7717; *SE* = 2.8324) did not differ from the 50kD-5 (*M* = 125.7108; *SE* = 4.1981), nor did the 75kD-5 (*M* = 123.5677; *SE* = 3.6294) differ from the 75kD-I (*M* = 130.7717; *SE* = 2.8324), *p* > .05. However, the 100kD-I injury (*M* = 147.9892; *SE* = 4.6903) resulted in higher DCF fluorescence than the 75kD-5 injury (*M* = 123.5677; *SE* = 3.6294), p = .043. The 100kD-5 injury (*M* = 168.8455; *SE* = 6.1463) also failed to cause significantly higher levels of DCF fluorescence compared to the 100kD-I (*M* = 147.9892; *SE* = 4.6903), p > .05. However, both the 150kD force groups resulted in increased DCF fluorescence compared to the previous injury treatment group, 150kD-I (*M* = 226.8258; *SE* = 15.5943) vs. 100kD-5 (*M* = 168.8455; *SE* = 6.1463) and 150kD-5 (*M* = 266.9611; *SE* = 17.4814) vs. 150kD-I (*M* = 226.8258; *SE* = 15.5943), *p* < .001. **(B)** There was a significant main effect of Force on DCF fluorescence in the CA1 region, *F*(3, 348) = 91.012, *p* < .001 (Figure 7B). *Post hoc* analyses (Fisher’s LSD) show that all levels of force, 50kD (*M* = 122.9371; *SE* = 2.4982), *p* = .029, 75kD (*M* = 127.1697; *SE* = 2.3229), *p* = .009, 100kD (*M* = 158.4173; *SE* = 4.0164), *p* < .001, and 150kD (*M* = 246.6394; *SE* = 11.8417), *p* < .001, caused increased DCF fluorescence compared to the Control group (*M* = 100.00; *SE* = 1.9495). Additionally, while the 75kD (*M* = 127.1697; *SE* = 2.3229), force did not result in more DCF fluorescence compared to the 50kD injury group (*M* = 122.9371; *SE* = 2.4982), *p* > .05, the 100kD group (*M* = 158.4173; *SE* = 4.0164) showed significantly higher DCF fluorescence than the 75kD group (*M* = 127.1697; *SE* = 2.3229), *p* < .001, and the 150kD group (*M* = 246.6394; *SE* = 11.8417) showed higher DCF fluorescence than the 100kD group (*M* = 158.4173; *SE* = 4.0164), *p* < .001. **(C)** There was a significant main effect of Duration on DCF fluorescence in the CA1 region, *F*(1, 348) = 6.088, *p* = .014 (Figure 7C). Both the 0s (*M* = 156.8616; *SE* = 5.3624) and 5s (*M* = 170.6695; *SE* = 6.5906) durations showed an increased DCF fluorescence compared to the Control group (*M* = 100.00; *SE* = 1.9495), *p* < .001. The 5s duration also showed a significant increase in PI uptake compared to the 0s duration, *p* = .023. *different from Control; #different from previous injury group.


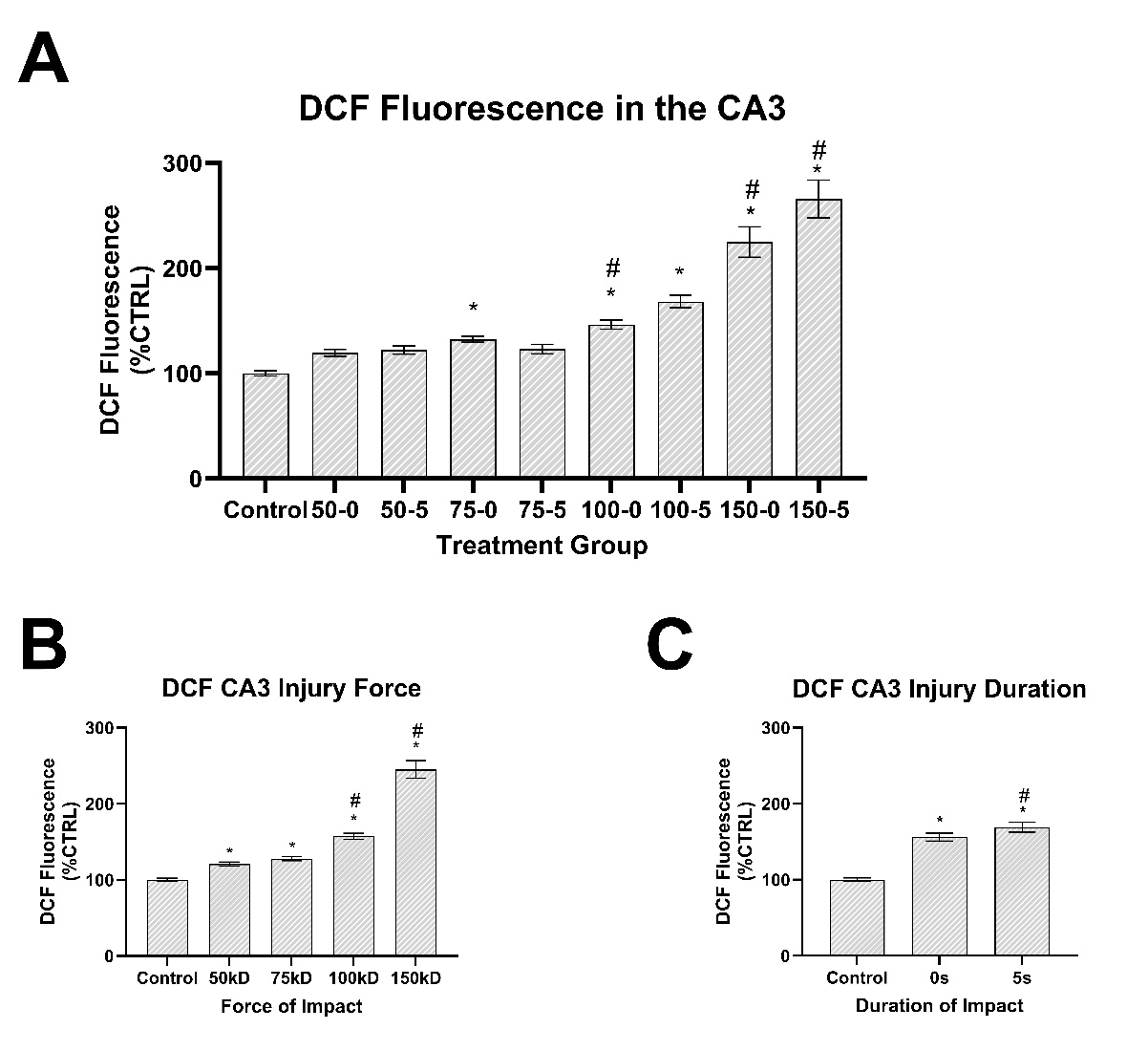
**Supplemental Figure 8.** There was no significant sex effect found in the CA3 region of the hippocampus, so data were collapsed across sex. **(A)** There was a main effect of Treatment in the CA3 region of the hippocampus, *F*(8, 348) = 42.289, *p* < .001 (Figure 8A). Fisher’s LSD comparison showed that neither duration for the 50kD force (0s *M* = 119.5404; *SE* = 3.2941 or 5s *M* = 122.3248; *SE* = 4.0223) resulted in higher DCF fluorescence compared to the Control (*M* = 100.00; *SE* = 2.3920). Interestingly, the long duration 75kD treatment group (*M* = 132.3881; *SE* = 2.8609) just failed to show a significant increase in DCF compared to Control (*M* = 100.00; *SE* = 2.3920), *p* > .05, however the 75kD-I group did (*M* = 132.3881; *SE* = 2.8609), *p* = .007. At both durations, the remaining forces of 100kD (0s *M* = 146.4799; *SE* = 4.2326 and 5s *M* = 168.3510; *SE* = 6.0877) and 150kD (0s *M* = 224.9637; *SE* = 14.5346 and 5s *M* = 265.8608; *SE* = 17.9305) did show increased DCF fluorescence compared to Control (*M* = 100.00; *SE* = 2.3920), *p* < .001. Post hoc analyses also tested if the level of injury caused significantly more than the previous injury level. The 50kD-5 (*M* = 122.3248; *SE* = 4.0223) did not differ from the 50kD-I (*M* = 119.5404; *SE* = 3.2941), the 75kD-I (*M* = 132.3881; *SE* = 2.8609) did not differ from the 50kD-5 (*M* = 122.3248; *SE* = 4.0223), nor did the 75kD-5 (*M* = 123.0909; *SE* =4.4603) differ from the 75kD-I (*M* = 132.3881; *SE* = 2.8609), *p* > .05. However, the 100kD-I injury (*M* = 146.4799; *SE* = 4.2326) resulted in higher DCF fluorescence than the 75kD-5 injury (*M* = 123.0909; *SE* =4.4603), p = .05. The 100kD-5 injury (*M* = 168.3510; *SE* = 6.0877) also failed to cause significantly higher levels of DCF fluorescence compared to the 100kD-I (*M* = 146.4799; *SE* = 4.2326), p > .05. However, both the 150kD force groups resulted in increased DCF fluorescence compared to the previous injury treatment group, 150kD-I (*M* = 224.9637; *SE* = 14.5346) vs. 100kD-5 (*M* = 168.3510; *SE* = 6.0877) and 150kD-5 (*M* = 265.8608; *SE* = 17.9305) vs. 150kD-I (*M* = 224.9637; *SE* = 14.5346), *p* < .001. **(B)** There was a significant main effect of Force on DCF fluorescence in the CA3 region, *F*(3, 348) = 91.429, *p* < .001 (Figure 8B). *Post hoc* analyses (Fisher’s LSD) show that all levels of force, 50kD (*M* = 120.9683; *SE* = 2.6015), *p* = .043, 75kD (*M* = 127.7395; *SE* = 2.6841), *p* = .007, 100kD (*M* = 157.4155; *SE* = 3.8838), *p* < .001, and 150kD (*M* = 245.1534; *SE* = 11.6690), *p* < .001, caused increased DCF fluorescence compared to the Control group (*M* = 100.00; *SE* = 2.3920). The 75kD (*M* = 127.7395; *SE* = 2.6841), force did not result in more DCF fluorescence compared to the 50kD injury group (*M* = 120.9683; *SE* = 2.6015), *p* > .05. However, the 100kD group (*M* = 157.4155; *SE* = 3.8838) showed significantly higher DCF fluorescence than the 75kD group (*M* = 127.7395; *SE* = 2.6841), *p* < .001, and the 150kD group (*M* = 245.1534; *SE* = 11.6690) showed higher DCF fluorescence than the 100kD group (*M* = 157.4155; *SE* = 3.8838), *p* < .001. **(C)** There was a significant main effect of Duration on DCF fluorescence in the CA3 region, *F*(1, 348) = 5.536, *p* = .019 (Figure 8C). Both the 0s (*M* = 156.3026; *SE* = 5.1275) and 5s (*M* = 169.3034; *SE* = 6.7005) durations showed an increased DCF fluorescence compared to the Control group (*M* = 100.00; *SE* = 2.3920), *p* < .001. The 5s duration also showed a significant increase in DCF fluorescence compared to the 0s duration, *p* = .03. *different from Control; #different from previous injury group.


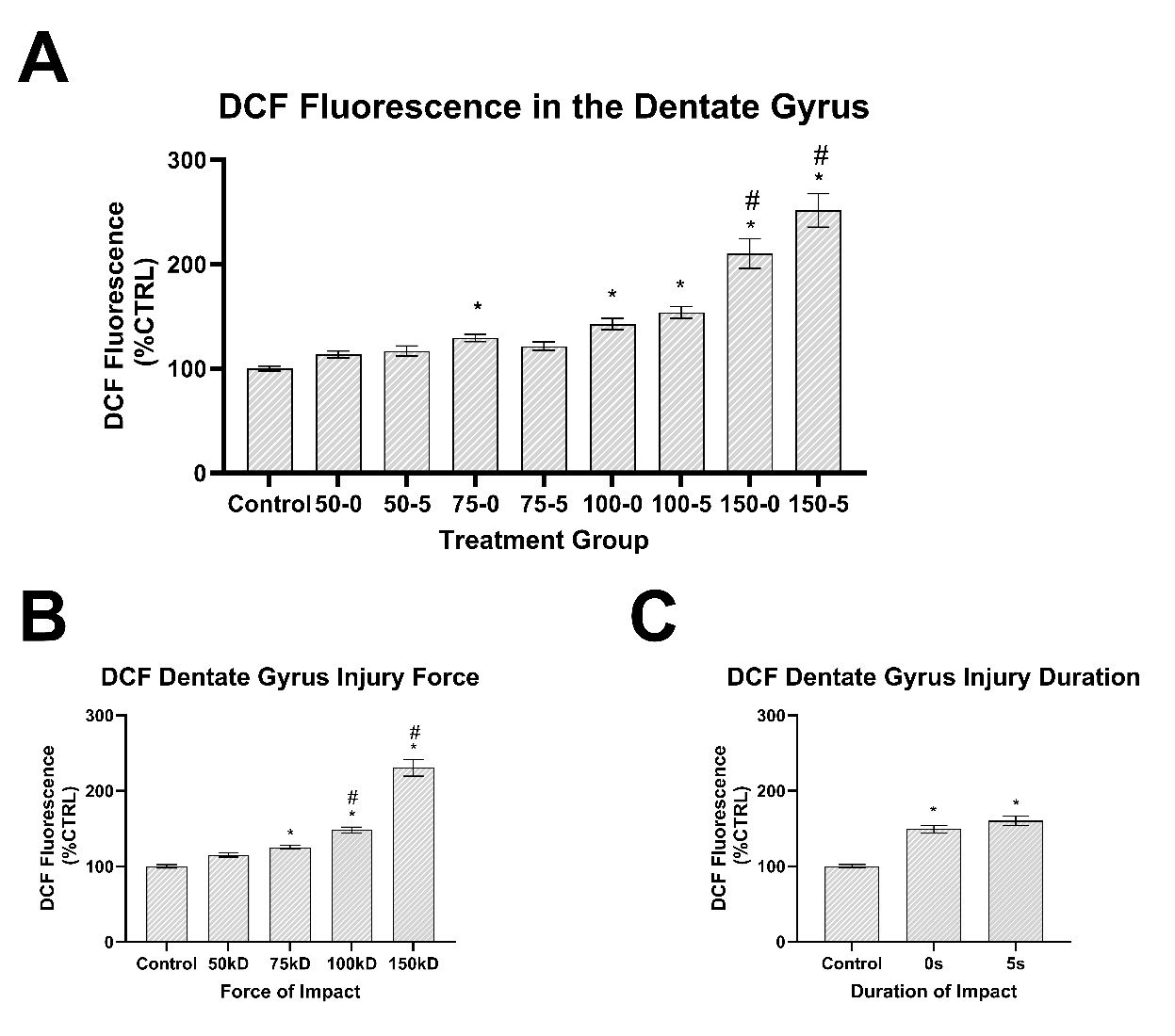
**Supplemental Figure 9.** There was no significant sex effect found in the DG region of the hippocampus, so data were collapsed across sex. **(A)** There was a main effect of Treatment in the DG region of the hippocampus, *F*(8, 348) = 38.467, *p* < .001 (Figure 8A). Fisher’s LSD comparison showed that neither duration for the 50kD force (0s *M* = 113.6774; *SE* = 3.1781 or 5s *M* = 116.9236; *SE* = 4.7075) resulted in higher DCF fluorescence compared to the Control (*M* = 100.00; *SE* = 2.4806), p > .05. The 5s duration 75kD treatment group (*M* = 121.4695; *SE* = 4.1772) just failed to show a significant increase in DCF compared to Control (*M* = 100.00; *SE* = 2.4806), *p* > .05, however the 75kD-I group did (*M* = 129.2675; *SE* = 3.4590), *p* = .010. At both durations, the remaining forces of 100kD (0s *M* = 142.7266; *SE* = 5.4138 and 5s *M* = 153.8102; *SE* = 5.6352) and 150kD (0s *M* =210.2599; *SE* = 14.2044 and 5s *M* = 251.5413; *SE* = 16.0689) did show increased DCF fluorescence compared to Control (*M* = 100.00; *SE* =2.4806), *p* < .001. *Post hoc* analyses also tested if the level of injury caused significantly more than the previous injury level. The 50kD-5 (*M* = 116.9236; *SE* = 4.7075) did not differ from the 50kD-I (*M* = 113.6774; *SE* = 3.1781), the 75kD-I (*M* = 129.2675; *SE* = 3.4590) did not differ from the 50kD-5 (*M* = 116.9236; *SE* = 4.7075), nor did the 75kD-5 (*M* = 121.4695; *SE* = 4.1772) differ from the 75kD-I (*M* = 129.2675; *SE* = 3.4590), *p* > .05. The 100kD-I injury (*M* = 142.7266; *SE* =5.4138) did not differ from 75kD-5 injury (*M* = 121.4695; *SE* = 4.1772) and the 100kD-5 injury (*M* = 153.8102; *SE* = 5.6352) also failed to cause significantly higher levels of DCF fluorescence compared to the 100kD-I (*M* = 142.7266; *SE* = 5.4138), p > .05. However, both the 150kD force groups resulted in increased DCF fluorescence compared to the previous injury treatment group, 150kD-I (*M* = 210.2599; *SE* = 14.2044) vs. 100kD-5 (*M* = 153.8102; *SE* = 5.6352) and 150kD-5 (*M* = 251.5413; *SE* = 16.0689) vs. 150kD-I (*M* = 210.2599; *SE* = 14.2044), *p* < .001. **(B)** There was a significant main effect of Force on DCF fluorescence in the DG region, *F*(3, 348) = 84.291, *p* < .001 (Figure 9B). The three highest forces, 75kD (M = 125.3685; SE = 2.7300), p = .010, 100kD (M = 148.2684; SE = 3.9321), p < .001, and 150kD (M = 230.6393; SE = 10.8923), p < .001, all caused increased DCF fluorescence compared to the uninjured Control (M = 100.00; SE = 2.4806). The lowest force, 50kD (M = 115.3421; SE = 2.8556), failed to show a significant increase in DCF. The 75kD (*M* = 125.3685; *SE* = 2.7300), force did not result in more DCF fluorescence compared to the 50kD injury group (*M* = 115.3421; *SE* = 2.8556), *p* > .05. However, the 100kD group (*M* = 148.2684; *SE* = 3.9321) showed significantly higher DCF fluorescence than the 75kD group (*M* = 125.3685; *SE* = 2.7300), *p* = .005, and the 150kD group (*M* = 230.6393; *SE* = 10.8923) showed higher DCF fluorescence than the 100kD group (*M* = 148.2684; *SE* = 3.9321), *p* < .001. **(C)** There was a significant main effect of Duration on DCF fluorescence in the DG region, *F*(1, 348) = 4.392 *p* = .037 (Figure 9C). Both the 0s (*M* = 149.4297; *SE* = 4.9507) and 5s (*M* = 160.3663; *SE* = 6.1686) durations showed an increased DCF fluorescence compared to the Control group (*M* = 100.00; *SE* = 2.4806), *p* < .001. The 0s and 5s duration did not significantly differ from each other, p > .05. *different from Control; #different from previous injury group.


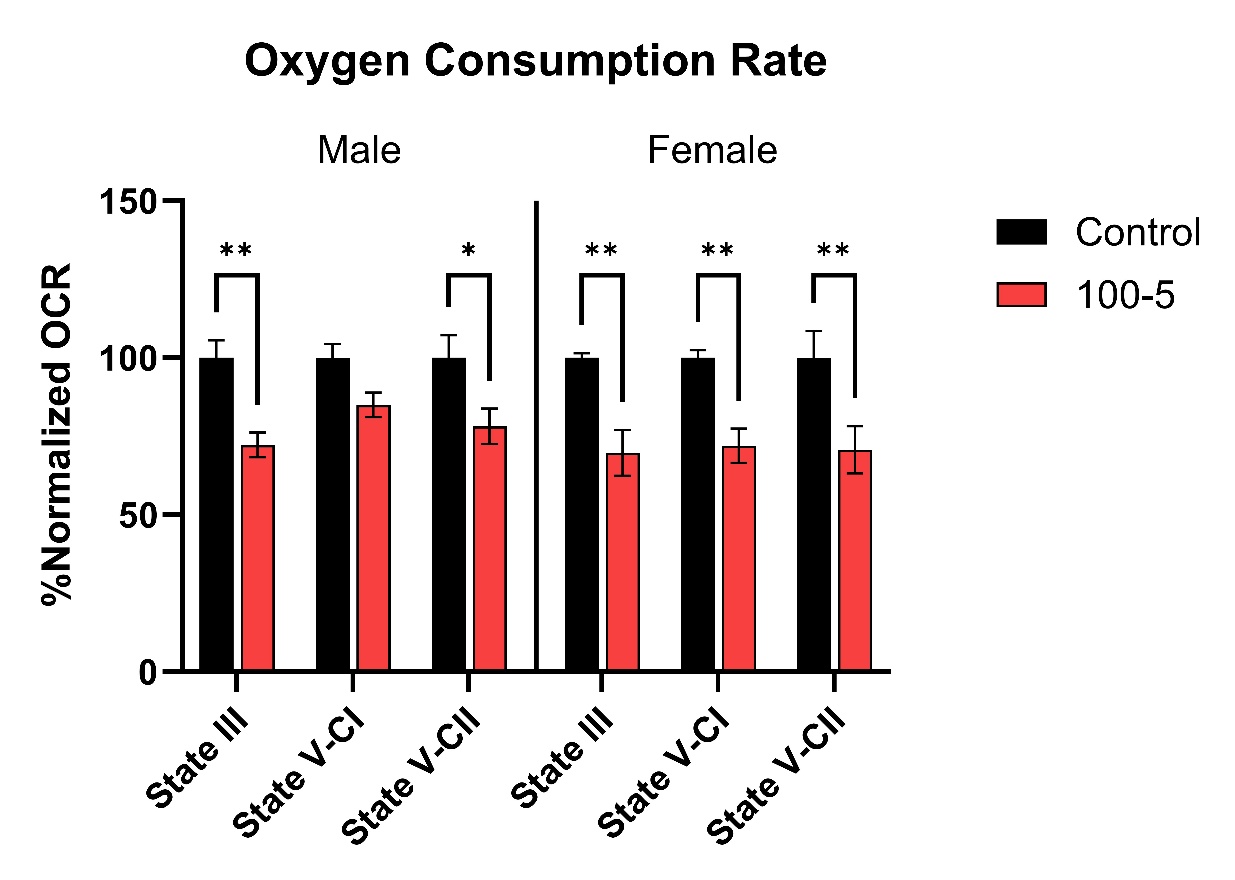


**Supplemental Figure 10.** The 100kD-5s injury disrupted mitochondrial bioenergetics in State III *p*-adj = .0071 and State V-CII *p*-adj = .0495 in male slices. Further, the 100kD-5s injury disrupted mitochondrial bioenergetics in State III *p*-adj = .0016, State V-CI *p*-adj = .004, and State V-CII *p*-adj = .0023 in female slices. There were no differences between sexes in the injured mitochondria across any of the assay States. N = 23 (11 males). Two-way ANOVA with Tukey’s *post-hoc*. **p* <.05, ***p* <.01.
